# Effectiveness of Dietary Supplement for Skin Moisturizing in Healthy Adults: A Systematic Review and Meta-Analysis of Randomized Controlled Trials

**DOI:** 10.3389/fnut.2022.895192

**Published:** 2022-06-02

**Authors:** Qian Sun, Jingping Wu, Guofei Qian, Hongbin Cheng

**Affiliations:** ^1^Department of Medical Cosmetology, Hospital of Chengdu University of Traditional Chinese Medicine, Chengdu, China; ^2^Haisco Pharmaceutical Group Company Ltd., Chengdu, China; ^3^Dermatology Department, Hospital of Chengdu University of Traditional Chinese Medicine, Chengdu, China

**Keywords:** skin moisturizing, dietary supplement, oral moisturizers, randomized controlled trials, meta-analysis

## Abstract

**Background:**

The dietary supplement industry offers many oral cosmetics that purportedly assist in skin moisturization often with unclear evidence supporting efficacy and safety. To update the accessible proofs pertaining to the safety and effectiveness of oral dietary supplements to facilitate skin moisturizing via an all-around review and meta-analysis.

**Methods:**

Three on-line databases [Pubmed, Embase, and Cochrane Library (CENTRAL)] were retrieved from January 2000 to November 2021. An overall 66 randomized controlled trials (RCTs) of skin care were recognized. Meta-analysis was performed for dietary supplements with four or more available research.

**Results:**

Oral collagen or ceramide resulted in a statistically significant increase in skin hydration and a decrease in transepidermal water loss (TEWL) compared to placebo. No benefits regarding the improvement of skin conditions in terms of water content and TEWL were observed for lactic acid bacteria or Lactobacillus fermented foods. A statistically significant and positive effect on skin hydration was observed for both hyaluronan and procyanidin, with an unknown effect on TEWL due to insufficient RCTs. There was a non-significant improvement in the water content of stratum corneum for astaxanthin based on subgroup analyses. Among the dietary supplements trialed in ≤ 3 RCTs, the judgment regarding their effects on skin moisturizing was prevented by inconsistent conclusions as well as insufficient research. All food supplements were safe throughout the research (normally ≤ 24 weeks).

**Conclusion:**

Oral dietary supplements, including collagen, ceramides, hyaluronan, and procyanidin, were proven to be effective for skin moisturization. At present, for skin moisturization, the proofs supporting the recommendation of other dietary supplements, such as lactic acid bacteria and astaxanthin, are insufficient.

**Systematic Review Registration:**

http://www.crd.york.ac.uk/PROSPERO/ identifier CRD42021290818

## Introduction

Keeping the skin tender and beautiful is our common wish, especially for females. Skin dryness highlight wrinkles, making an individual look older. As the largest organ of the human body, the skin serves as a barrier between the cells and the external environment, covering most of the human body (1). The water content (10% to 20%) of the stratum corneum is essential to maintain the integrity of the structure and function of the skin barrier system, which plays a decisive role in skin health (2). Skin barrier dysfunction due to environmental factors or diseases leads to skin dryness, roughness, and chapped skin, which is also a most commonly seen skin problem, especially in winter (3, 4). Topical moisturizers, as the most widely used components of basic skin care (5), increase skin hydration by preventing water loss from the skin surface, which is known as “moisturizing from the outside.” Moisturizers are safer in contrast to conventional medicines utilized by doctors (6, 7). Nevertheless, uncomfortable skin reactions from topical preparations, like sensory reactions or subjective sensations (no signs of inflammatory events), may still be encountered after application (7–9). For example, facial skin, which exhibits more sensitivity in contrast to other body regions often, suffers from smarting, burning, and stinging sensations among individuals using dermatological preparations. Meanwhile, the skin, as living tissue, requires nourishment to keep itself healthy (10). The needed nutrients are predominantly derived from foods rather than merely topical preparations.

Over the past 20 years, the consciousness to realize “beauty from within” has been growing. Hence, for the purpose of maintaining a healthy lifestyle, people begin to ingest nutrients from foods (11). Therefore, approaches based on dietary supplements or functional food, known as “moisturizing from the inside”, have aroused more and more interest. Actually, a current innovation in the field of skin care is the intake of dietary supplementations and the incorporation of the use of topical formulations, which are proposed to improve both the inner and outer appearance of the skin, aiming to restore the deepest skin layers and ameliorate surface appearance radically (7, 12). Therefore, here is the question: among substantial dietary supplements, which one can assist in maintaining skin water content and sustaining skin integrity?

Dietary supplements are defined as the dietary components as follows: vitamins, minerals, herbs, and other plant extracts, amino acids (AA), dietary substances utilized to elevate the overall daily intake of food nutrients, or a concentrate, metabolite, ingredient, extract or combination product of the aforesaid substances (13). Maintaining good nutritional status via intaking micronutrients, macronutrients and other bioactive constituents are pivotal for skin health and appearance. Substantial food supplements have been reported to exert beneficial effects on whitening, anti-wrinkle performances, and the removal of wrinkles and freckles, contributing to a more youthful appearance (10, 11, 14). However, reviews on the investigation of individual dietary supplements for skin moisturizing are insufficient, even if it's vital for skin care. Moreover, the results obtained from many RCTs evaluating skin hydration by the same food supplement are confusing and even contradictory, causing trouble for consumers. The scientific evidence pertaining to skin moisturized by dietary supplements are often scarce and are usually derived from *in vitro* or uncontrolled *in vivo* studies.

Despite the substantial available supplements, few have enough, compelling and scientific validation regarding their efficacy and safety. Different from pharmaceutic medicines requiring approval prior to commercial application, dietary supplements do not require clinical evidence. With the increasing number of scientific publications and clinical studies aiming at realizing beauty from within, it is necessary to compile and analyze these data and assist in decision-making concerning dietary supplements. Hence, the present study will review what is currently known about dietary supplements with regard to skin moisturizing, focusing on the potency and safety via a comprehensive review and meta-analysis of RCTs.

## Methods

A review protocol was registered in advance in the PROSPERO database (CRD42021290818). The present paper was completed as per the PRISMA (Preferred Reporting Items for Systematic Reviews and Meta-Analyses) guideline involving recognition, selection, eligibility, and inclusion (15).

### Search Strategy

Three on-line databases were retrieved from January 2000 to November 2021, as oral dietary supplements for skin health have only been extensively studied over the last 20 years according to the “results by year” function of Pubmed. The literature retrieval was finished via search terms as follows: 1 skin hydration or skin conditions or skin aging; 2) dietary supplements. These search terms were adjusted for every database accordingly. The retrieval method was fully presented in Supplementary Table S1. Merely English language RCTs were selected herein.

### Eligibility Criteria

Research was selected when it satisfied every inclusive criterion below:

(1) Types of studies. Merely parallel-group randomized controlled trials (RCTs) that evaluated skin conditions including hydration or TEWL conducted in human subjects were selected. This type of trial can decrease biases via randomization and the parallel-group design to control participant differences and they can introduce a control group that takes a placebo supplementation or a lower dose of a supplement to eliminate the placebo effect. Then, published papers must be peer-reviewed articles with full text.

(2) Types of participants. Healthy individuals of any gender or race, aged between 18 and 75 years were included. The main exclusion criteria were as follows: (a) pregnancy, breastfeeding, and various metabolic, cardiovascular, or hepatic diseases; (b) serious skin related diseases such as atopic dermatitis, psoriasis, and vitiligo; (c) regular consumption of any food, medicine, or other supplements that affect skin conditions, including health food, antioxidant supplements, retinoids, steroids, and any other hormonal products.

(3) Types of interventions. RCTs comparing dietary supplements with a placebo or the lower dose of the same dietary supplement were eligible. A Study was eligible regardless of any extra life-style interventions utilized when the identical regimen was utilized in the test and the control groups. Research using a diverse regimen between the test group and placebo group were discarded.

(4) Number of researches for each dietary supplement. Only when there were at least two studies investigating the same dietary supplement via assessing either of the two outcomes (skin hydration and TEWL) would we include these researches in this systematic review.

### Study Selection

Two reviewers (Q.S. and G.Q. or J.W.) screened titles and abstracts in an independent way. Full texts were imported into Endnote (Version X9, Clarivate Analytics) when researchers probably satisfied the inclusion criteria. Those full texts were examined by two researchers (Q.S. and G.Q. or J.W.) in an independent manner and evaluated against the aforesaid principles for an eventual determination regarding the eligibility.

### Data Extraction

The features of every research were listed in Supplementary Table S2, involving research details like (author, nation and year of publication), research populations (age), sex, and health status), study duration (weeks), intervention content of each group, daily dosage (mg), the form of applications, testing environment [°C room temperature (R.T.) and % relative humidity (R.H.)], starting and ending times of the research, measurement instruments, measuring sites in the skin and units of measurement. Safety was evaluated between the test group and placebo group against adverse events (AE), treatment-associated adverse effects (TAE), and treatment-associated withdrawals (TAW).

Other harvested data, like the baseline and endpoint measure (average) and its variability SD (standard deviation) or SE (standard error), were abstracted by GetData Graph Digitizer when the research merely displayed outcomes as figures (16). Those values with defined units for the results were introduced into an Excel spread sheet. Two outcomes were captured: moisture content and TEWL. The test areas on faces and arms valued by women were preferred as the measurement areas to evaluate moisturizing effects when studies provided data from multiple measurement sites. The data of the supplementation with the longest observation duration were extracted for certain screened researches which carried out result measurements at multiple temporal points.

### Quality Assessment

Risk of bias (ROB) assessed by the normal ROB evaluation tool for RCTs, according to the recommendation by the Cochrane Collaboration (17), was independently examined by two reviewers (Q.S. and G.Q. or J.W.). Such tool evaluates ROB in seven items including selection biases (SB) (stochastic sequence generation and allocation concealment), performance biases (PB) (blinding of subjects and researchers), detection biases (DB) (blinding of result evaluators), attrition biases (AB) (non-complete result data), reporting biases (RB) (selective report), and the rest of biases which involve other main imperfections in the trial designs or methods (e.g., changed life habits during the study, unstable conditions of test rooms badly influencing the measurement results and potential conflicts of interests). These seven criteria were assessed in each study, generating a gradation of “low risk,” “high risk” or “not clear.” A criterion was at a low ROB when the study adequately reported methods without potential bias, based on the Cochrane Collaboration's guideline (17). Similarly, a high ROB was assigned to the trial for a criterion when the research described a method unable to remove underlying biases. Otherwise, the unclear risk of bias would be assigned to the trial, if there wasn't information or inadequate information.

### Statistical Analysis

#### Evaluation of Overall Effect Size

The RCTs were grouped according to the types of dietary supplements, and Review Manager 5.4 was independently utilized to finish meta-analysis for each group (18). Within each group, studies would be further categorized into subgroups including single-agent preparation and combination preparation if there were more than one potential active ingredient in the test food compared to the placebo. When there were at least four studies of a dietary supplement via assessing either of the two outcomes, meta-analysis was conducted.

The standardized mean differences (SMDs) for continuous variables were calculated by means of the random-effect model applied in statistical analysis. Mean difference was achieved by the value at the endpoint subtracted from the value at baseline. If standard deviations (SDs) variations from the baseline were not reported, they would be calculated based on SDs values for the baseline and final point in both the treatment group and the placebo group by the formula:


$$\begin{array}{l} SD\;=\;\sqrt{SD_{baseline}^{2}+\;SD_{final}^{2}-\;2\;\times \;Corr\;\times \;SD_{baseline}\;\times \;SD_{final}} \end{array}$$

Because the baseline–final correlation coefficients (Corr) were not mentioned in these researches, a Corr value of 0.5 was utilized (19), based on calculated values in most articles with sufficient data ranging from 0.4 to 0.6. As for cases reported as the SE of the mean (SEM), SD was determined by $SD=SEM\;\times \sqrt{n}$ (n is the number of participants) (19, 20).

In addition, if an identified research had several contrasts (groups/treatment arms) between intervention and placebo, for instance, low dosage and high dosage groups or face-measurement and arm-measurement groups, then each comparison would be identified as a separate trial. The sample size of the intervention group was divided equally among the contrasts to prevent the double counting of the participants and subsequent unit-of-analysis error. If RCTs did not have a placebo group for comparison and only compared the effects of low dose intervention to high dose intervention, the high dose group would be considered the test group and the low dose group would be the placebo group. Both the effect sizes of moisture content and TEWL were expressed as SMDs with 95% confidence intervals (CIs) because not all studies reported these two outcomes measured by the same equipment and in the same unit. A *p* < 0.05 had significance on statistics.

#### Evaluation of Heterogeneity

Heterogeneous statistics were estimated by Higgins I (I^2^) in the legend of every figure with a forest plot. The I^2^ index represents the proportion of the observed differences in effect sizes owing to inhomogeneity instead of sampling errors in total variation between studies (21). For example, an I^2^ of 0% represents that the entire identified variations in effect sizes result from sampling errors and an I^2^ of 100% represents that the entire identified variations in effect sizes result from variability in the true effect. I^2^ values of 0–25%, 25–50%, 50–75%, and 75–100% are normally regarded as low, moderate, high, and remarkably high heterogeneity, separately (22).

## Results

### Identification of Researches

**Figure 1:**
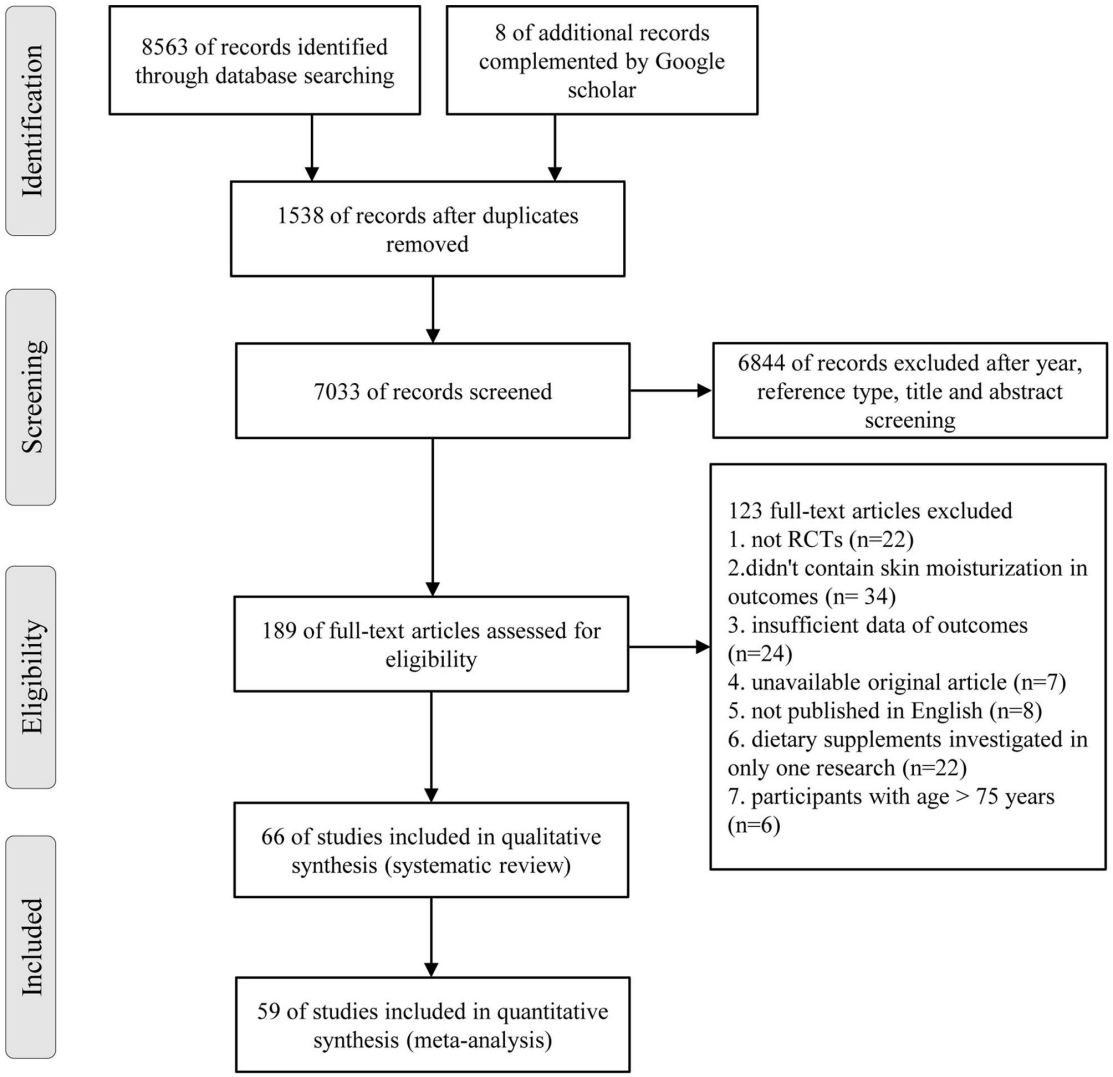
Flow diagram for study selection process.

An overall 8,563 references were recognized (Figure 1), involving 1,729 recognized by Pubmed, 5,434 by Embase, 1,400 by Cochrane Library, and 8 complemented by Google scholar. After the screening process, 66 RCTs satisfied the inclusive requirements.

### Risk of Bias Within Researches

**Figure 2:**
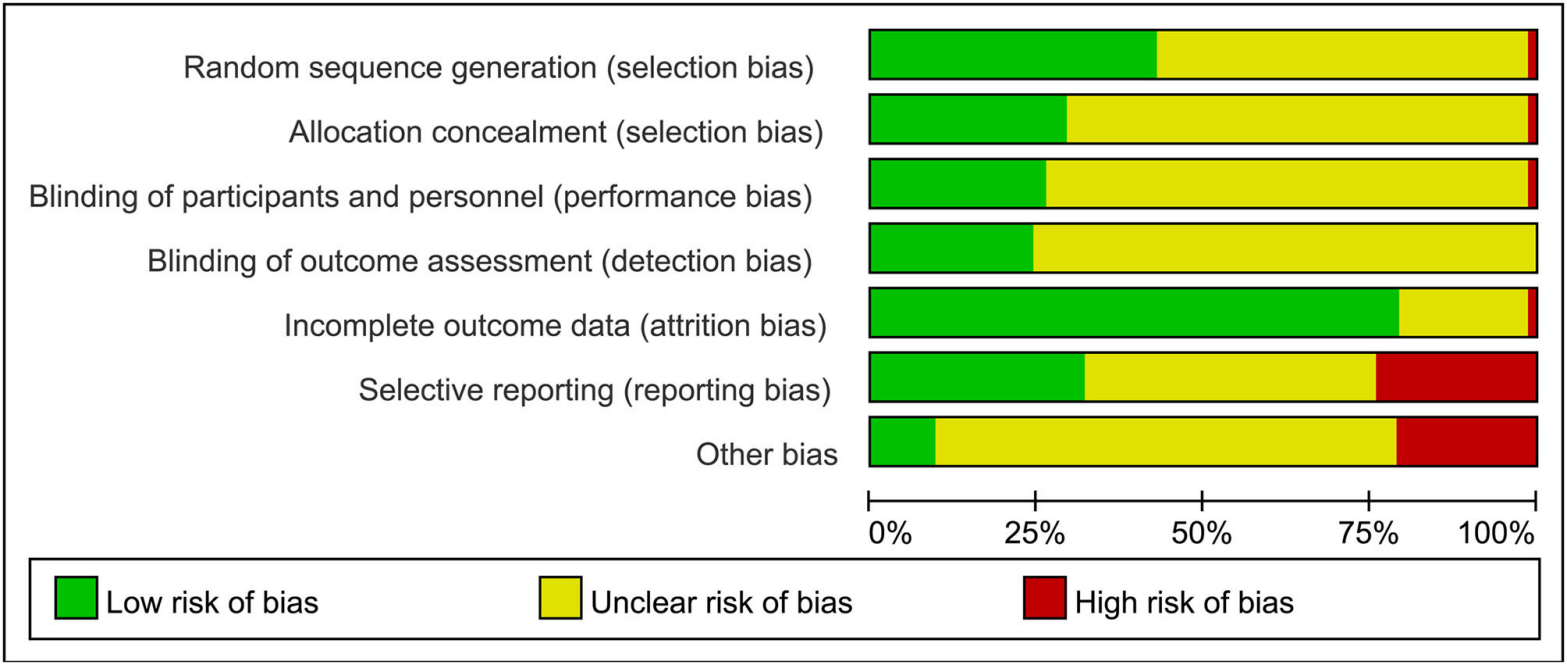
Summary of review authors' judgements for each risk of bias domain.

The review of every ROB item presented as percentages across the entire selected research as per the judgment of the researcher was displayed in Figure 2, and the judgment for every trial was described in Supplementary Figure S1. Limited details in research designs and methods were provided in most trials. Hence, the risk of SB was mostly vague: merely 28 of 66 RCTs (42%) sufficiently mentioned randomization, and 1 research was considered to be at high risk. Only 19 RCTs (29%) reported adequate allocation concealment and 1 study was thought to have a high risk. PB was predominantly not clear (74%) with 17 research works noting the blinding process of subjects or researchers and 1 study identified as high-risk. DB was predominantly not clear (76%), with 16 research works identified as low-risk. AB was low in 52 research works (79%), and only 1 research was regarded as at high risk. The risk of reporting bias was unclear in 27 studies (41%), and 21 studies (32%) provided concrete data and pre-registered their trials in a publically available trial registry. Fourteen research works (21%) were rated at a high risk of “other biases.” Commonly seen causes involved potential clashes of interests, the maintenance of participants' living habits, and insufficient details in physical environmental conditions for hydration measurement.

### Research Features

Supplementary Table S2 summarized the key features of each study. The most frequently investigated dietary supplements were Collagen (*n* = 16, 24%), Lactic acid bacteria (*n* = 14, 21%), Ceramides (*n* = 11, 17%), Polyphenols (*n* = 9, 14%), Carotenoids (*n* = 7, 11%) and Hyaluronan (*n* = 4, 6%). Fifty-nine of 66 randomized controlled studies were classified and then gathered into a meta-analysis to evaluate the roles of oral dietary supplements in skin moisturizing. Among the 59 trials gathered into a meta-analysis, 41 were conducted in Asian countries including China, Japan, Korea, and Thailand. The major participants were women with defined skin aging signs like wrinkles and dryness at baseline, aged between 30 and 60. The formulations of test products and placebo included capsules, beverages, and tablets in most trials lasting for 4 to 12 weeks. Furthermore, as physical environmental conditions are crucial for measurement, the majority of the research works were conditioned to the average values of relative R.T. (22 °C) and R.H. (50%).

### Collagen

Collagen was explored in 16 research works (*n* = 931), of which 10 focused on single-collagen products (23–32) and six of which highlighted combination preparation (CP) (33–38). The active ingredients involved in the studies included collagen peptides or hydrolysates with molecular weights ranging from 500 to 5,000 Da in 15 RCTs and sea fish cartilage that is rich in collagen in the RCT of Primavera 2005. Fish (8 RCTs) was a major source of Collagen besides porcine (1 RCT) and bovine (1 RCT) in the studies that provided origins of test products. The dosages of collagen ranging from 1 to 10 g daily were reported in single-collagen studies and the dosages ranging from 0.3 to 10 g daily were reported in combination preparations studies, except for the RCT of Primavera 2005 (36) in which there was no related description. No resemblance existed between the CP formulations. The research duration was between 6 and 12 weeks.

More than half of the studies adequately reported randomization including a computer-generated randomization schedule and random number table, while the research of Bolke 2019 (33) was at a high risk of SB for alternating allocation of subjects, and the research of Choi 2014 (24) was at high risks of SB and PB for the failure to blind the patients or therapists. The judgment regarding the risks of SB, PB, and DB was largely prevented by insufficient reporting of trial designs and methods across the research. Thirteen studies were at low risk of attrition bias because they had low dropout rates and reported reasons for exclusion. Four researches were at high risk of reporting bias, as their trial registrations were absent, and merely graphic representations or differences from baseline to endpoint were provided and no absolute values were reported. As it was impossible to judge whether or not the participants maintained their living habits or if physical environmental conditions for measurement were relatively stable, ten researches were at unclear risks of other biases, and two studies were at high risks of other biases due to the underlying clashes of interests because certain researchers were employees of corporations that owned the relevant dietary supplements.

**Figure 3:**
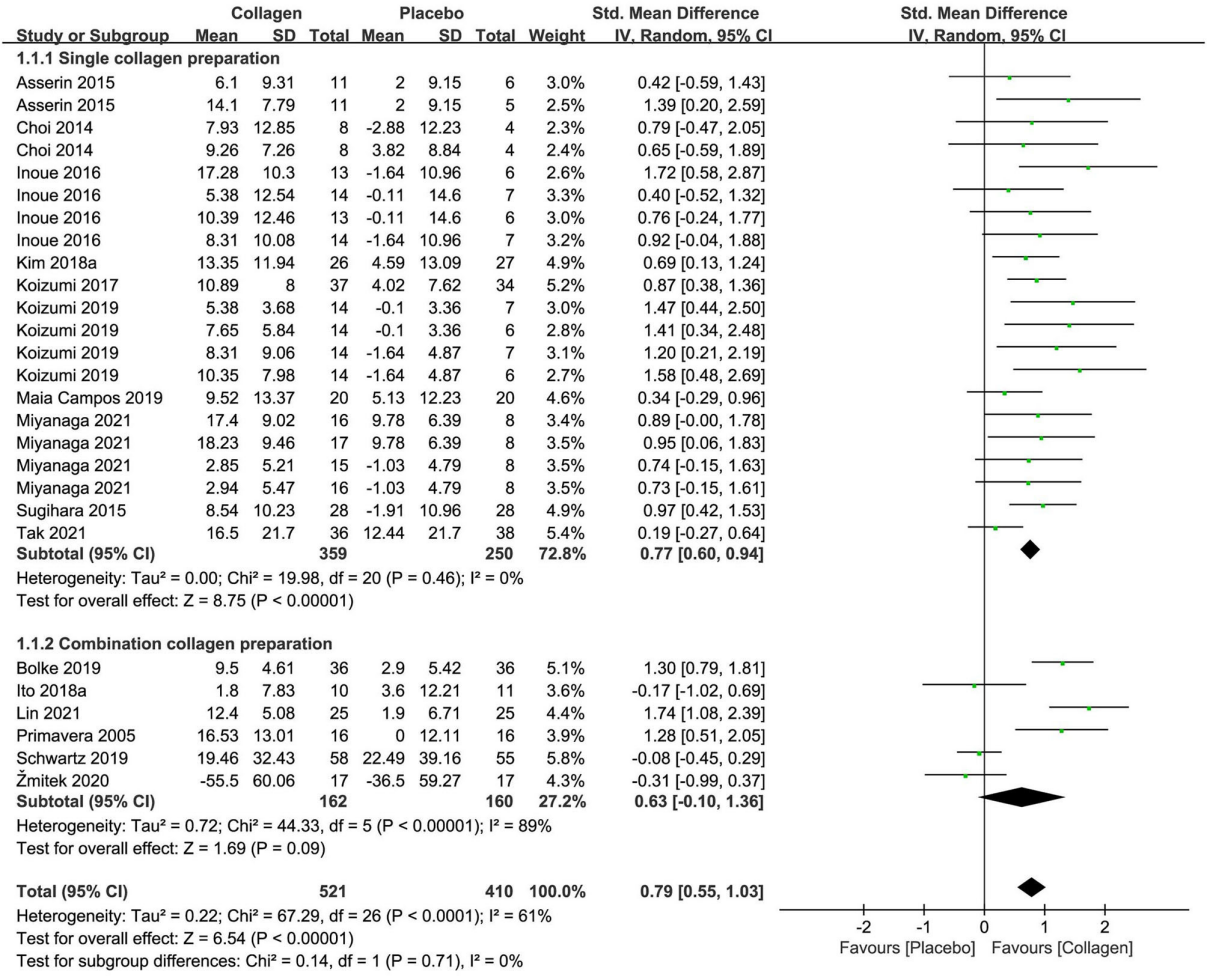
Forest plot of comparison: Collagen vs. placebo on skin hydration (SMD). The details of each study are reported in Supplementary Table S2. CI, confidence interval.

The forest plot of the meta-analysis of 16 researches is displayed in Figure 3 regarding combined moisture content estimates between the intervention and placebo groups. The meta-analysis of 10 researches (*n* = 609) of collagen as a single preparation revealed remarkable effects on skin hydration, with an SMD + 0.77 (95% CI +0.60, +0.94; *p* < 0.00001) in contrast to placebo. However, the combination preparations containing collagen exhibited unremarkable effects (SMD +0.63 95% CI −0.10, +1.36; *p* = 0.09) in contrast to placebo in 322 subjects. As a whole, the single-collagen research displayed no inhomogeneity (I^2^ = 0%), but research investigating CP exhibited remarkable inhomogeneity (I^2^ = 89%) for reasons including multiple active ingredients and different methods of measurement. Even so, on the foundation of the overall effect size (OES) of 0.79 (95% CI = 0.55, 1.03), the pooled analysis of studies for collagen supplements showed remarkable amelioration in skin hydration in terms of statistics (*p* < 0.00001).

In the meta-analysis with 6 trials (Supplementary Figure S2) that estimated transepidermal water loss (TEWL), oral collagen supplementation displayed a non-significant effect on the outcome (*p* = 0.05) in contrast to the placebo group, with an OES of −0.21 (95% CI = −0.42, 0.00). However, the single-collagen preparation was considered to have a statistically significant improvement according to the OES of −0.34 (95% CI = −0.63, −0.04; *p* = 0.02). Therefore, those discoveries ought to be treated with caution due to insufficient researches.

Safety was discussed in 11 of the 16 researches, and none of the 11 studies reported any adverse events due to the ingestion of collagen.

### Ceramides

Ceramides were examined in 11 researches (*n* = 601) over 6 to 20 weeks, nine of which focused on single-preparation products (39–47) and two of which highlighted combination preparation (36, 48). The active ingredients involved in the studies included wheat or rice polar lipids (5 studies), vegetable ceramides (3 studies), ceramide-containing acetic acid bacteria (2 studies), and ceramides from soy sauce lees (1 studies). Daily dosages of ceramides were discussed in eight researches within the range of 0.4 to 30 mg. The formulations of the CP did not display a resemblance.

An insufficient report in part of the researches made it hard to judge the risks of SB, PB, and DB. Nine researches were at low risk of AB (0–9.6% attrition) and one study was at high risk (31% attrition). As for reporting bias, six of the 11 studies were at unclear risk while three researches were at high risk because of the lack of trial registration numbers and the use of graphical representations or differences instead of absolute values. Two of the 11 researches were regarded as high risk of other biases on account of potential conflict of interest or both the lack of detail regarding environmental conditions for measurement and the uncertainty about whether or not participants maintained living habits.

**Figure 4:**
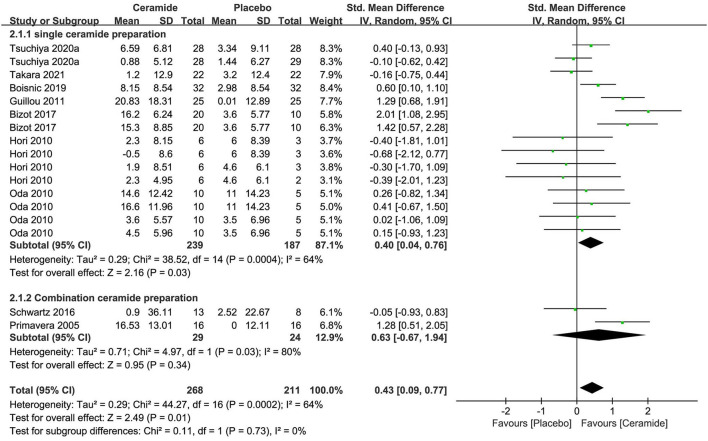
Forest plot of comparison: Ceramide vs. placebo on skin hydration (SMD). The details of each study are reported in Supplementary Table S2. CI, confidence interval.

The meta-analysis of seven researches (*n* = 426) exploring ceramides as single preparation displayed remarkable effects on improving the water contents of stratum corneum of skin in contrast to placebo [SMD 0.40; 95% CI 0.04, 0.76; *p* = 0.03 (Figure 4)]. The meta-analysis of two researches (*n* = 53) on CP involving ceramides displayed no obvious benefits on skin hydration with an SMD 0.63 (95% CI −0.67, 1.94; *p* = 0.34) in contrast to a placebo because of much fewer studies. In those 2 subgroups meta-analysis, the entire researches were remarkably heterogeneous (I^2^ = 64% and 80%, respectively) potentially because of the diversity of ceramides sources including cereal crop, soy sauce, vegetable, and acetic acid bacteria.

The meta-analysis was conducted in seven trials to evaluate TEWL (Supplementary Figure S3), and a consequence that oral ceramide supplement as single preparation remarkably ameliorated the result (*p* = 0.003) compared to the placebo group was achieved as per an OES of −0.29 (95% CI = −0.49, −0.10).

Eight of the 11 studies with respect to ceramides discussed safety. Six reported no AE, whereas two reported AE without treatment-related adverse effects or treatment-related withdrawal.

### Lactic Acid Bacteria

Lactic acid bacteria or Lactobacillus fermented food was explored in 14 researches (*n* =884), ten of which focused on single-preparation products (49–58) and four of which highlighted CP (59–62). Seven studies reported the daily dosages ranging from 1 × 10^10^ to 40 × 10^10^ cfu over 4 to 24 weeks, and two studies reported heat-killed Lactobacillus with doses between 25 and 50 mg every day over 8 to 12 weeks. No resemblance existed in the CP formulations apart from the researches of Kano 2013 (60) and Mori 2016 (61), as these two studies had similar ingredients.

Details in trial designs and methods were commonly inadequate in all researches, which made it hard to evaluate SB, PB, and DB. Ten researches were considered at low risk of AB (0–9.4% attrition). Four researches were identified to be at high risk of reporting bias due to a lack of details in trial registration and the absence of absolute values. Because certain researchers were employees of the corporation that owned relevant dietary supplements, the potential conflict of interest automatically became the major reasons for the high risk of other biases in three researches, and the rest were at unclear risk of other biases because there was no adequate information on their conflicts of interests or measuring environments or living habits.

**Figure 5:**
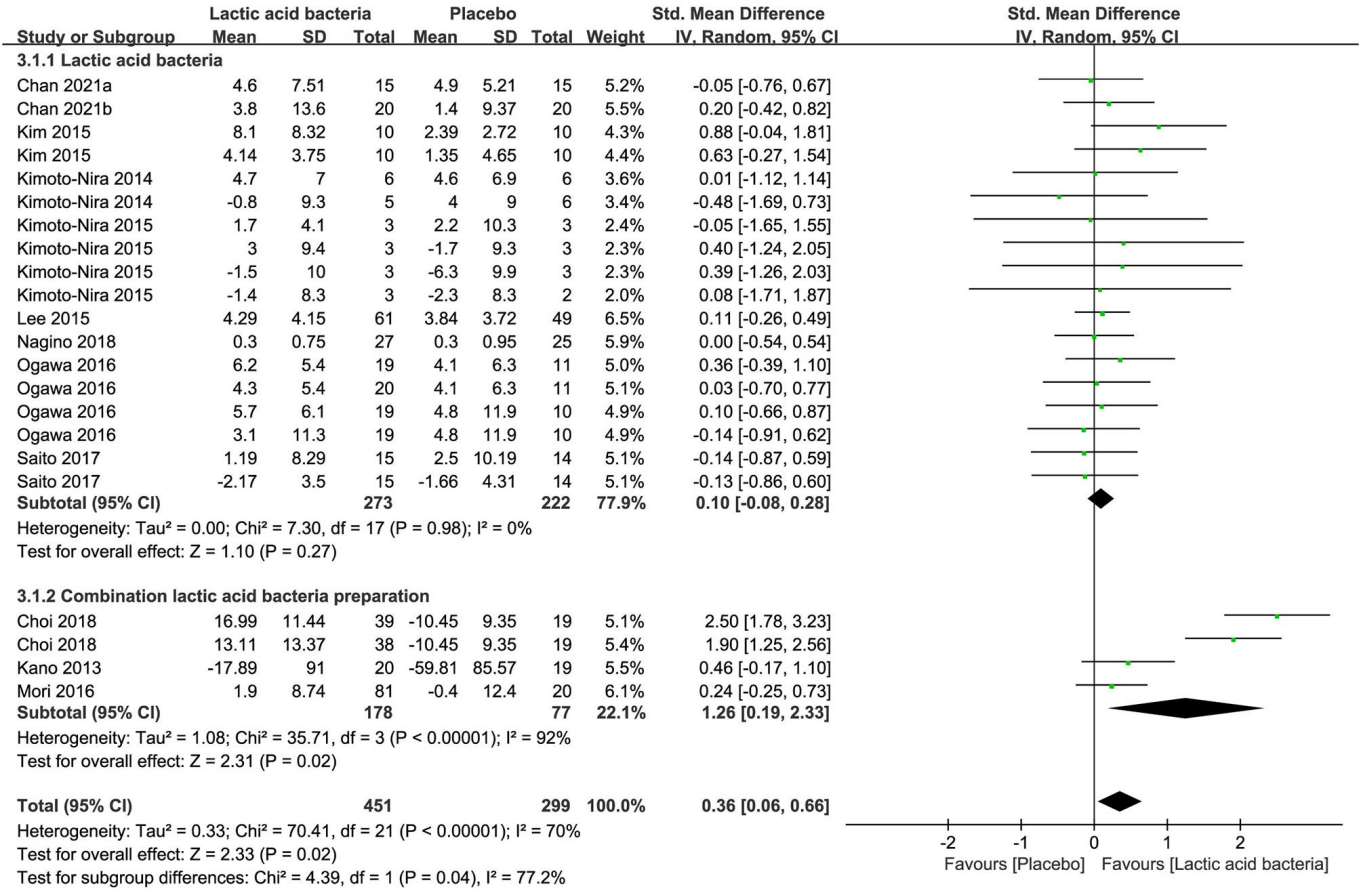
Forest plot of comparison: Lactic acid bacteria vs. placebo on skin hydration (SMD). The details of each study are reported in Supplementary Table S2. CI, confidence interval.

A non-remarkable effect on skin hydration was observed for lactic acid bacteria as a single preparation in contrast to placebo in 495 subjects of nine studies [SMD + 0.10; 95% CI−0.08, 0.28; *p* = 0.27 (Figure 5)]. Combination preparation containing Lactic acid bacteria, on the contrary, displayed remarkable effects on skin hydration (SMD + 1.26; 95% CI 0.19, 2.33; *p* = 0.02) in contrast to placebo in 255 subjects of three studies (Figure 5). It can be explained by the assumption that galacto-oligosaccharides (63) or honeybush (64) included in the combination preparation could produce benefits for skin moisturizing. Meanwhile, the single-preparation studies displayed a low heterogeneity in moisture content (I^2^ = 0%) and the researches on CP were remarkably inhomogeneous (I^2^ = 92%), which resulted from complicated components contained in the supplement products.

Furthermore, the forest plot (Supplementary Figure S4) of the meta-analysis of seven researches (*n* = 576) in reference to combined TEWL estimates between the placebo group and the intervention group also showed a similar effect that the single preparations generated non-remarkable effects on TEWL (SMD −0.10; 95% CI −0.30, 0.10; *p* = 0.33) in contrast to placebo in 389 subjects of five studies and the combination preparations had a statistically significant decrease in TEWL (SMD −0.45; 95% CI −0.75, −0.15; *p* = 0.004) in contrast to placebo in 187 subjects of two studies.

Nine of the 14 studies investigating Lactic acid bacteria mentioned safety, five reported no AE, whereas 3 reported no TAE, and 1 reported AE but no TAE.

### Hyaluronan

Hyaluronan was examined in four studies (65–68) (*n* = 196) as a single-preparation over 4 to 12 weeks. The daily dosages of hyaluronan were 120 mg in three RCTs and 200 mg in one RCT, respectively.

There were also no adequate details in trial designs and methods across the researches, which significantly exerted a negative impact on evaluating the risks of SB, PB, and DB. Three researches were at low risk of AB for the low dropout rates and detailed descriptions. Two researches were at unclear risk of AB for the lack of trial registration. The research of Hsu 2021 was at high risk of other biases, as two researchers were the employees of the corporation owing the dietary supplement.

**Figure 6:**
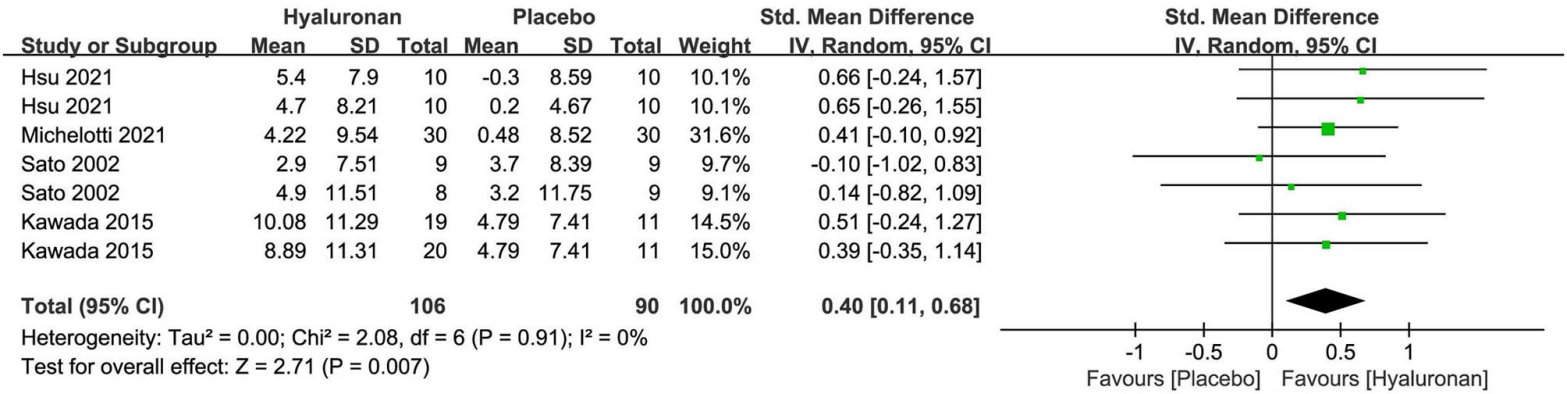
Forest plot of comparison: Hyaluronan vs. placebo on skin hydration (SMD). The details of each study are reported in Supplementary Table S2. CI, confidence interval.

The meta-analysis of four researches (*n* = 196) on hyaluronan as a single preparation generated remarkable effects on skin hydration, with an SMD + 0.40 (95% CI 0.11, 0.68; *p* = 0.007) in comparison to placebo (Figure 6). No researches containing hyaluronan as a combination preparation reported the effect on moisturization. For the outcome of TWEL, both the researches of Michelotti 2021 (67) and Hsu 2021 (65) reported that the HA group displayed significantly lower transdermal water loss in the face over 4 to 12 weeks of intervention. Non-remarkable differences in the arms versus the placebo group were revealed by the RCT of Hsu 2021, which requires further validation in the future.

All 4 studies mentioned the ingestion safety of HA. Three reported no AE and one reported no TAE.

### Polyphenols

Polyphenols were tested in nine researches (*n* = 495) as single-preparation products. The active ingredients involved in the studies included procyanidin (69–72) (4 studies), flavanol (73, 74) (2 studies), and coffee polyphenols (75, 76) (2 studies), and green tea polyphenols (77) (1 studies). The dosages of polyphenols were mentioned in eight researches within a range of 15 to 1,400 mg every day over 6 to 12 weeks.

Details in trial designs and methods were also commonly insufficient across the researches. Six researches were at low risk of AB (0–9.3% attrition). Two researches were at high risk of reporting bias for a lack of trial registration and the usage of graphical representations or differences instead of absolute values. The potential conflict of interest was the major reason for the high risk of other biases in both the RCTs of Shoji 2020 and Tsuchiya 2020b.

**Figure 7:**
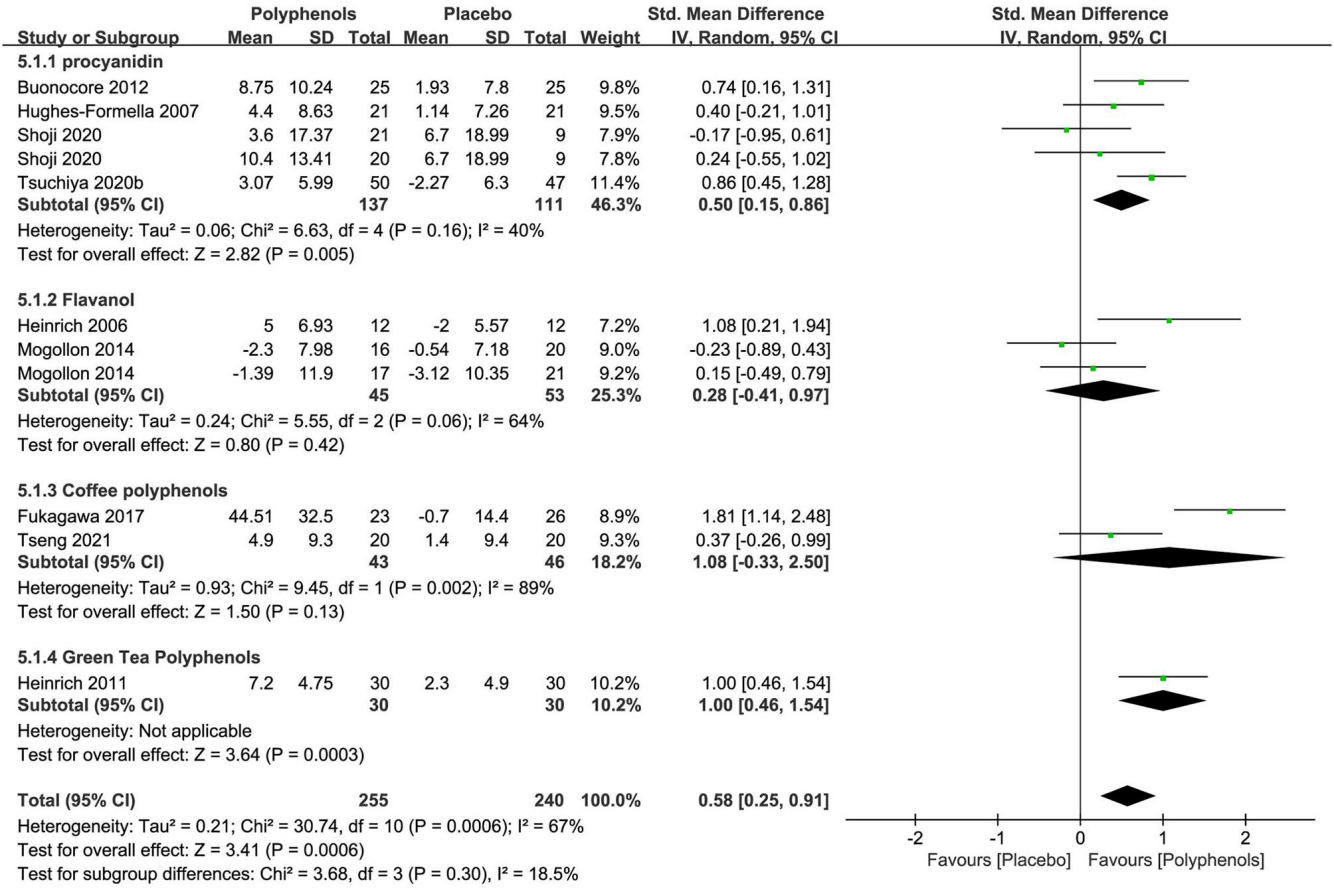
Forest plot of comparison: Polyphenol vs. placebo on skin hydration (SMD). The details of each study are reported in Supplementary Table S2. CI, confidence interval.

The subgroup meta-analysis of four researches (*n* = 248) exploring procyanidin as a single preparation displayed a significant effect on improving the stratum corneum water content in contrast to placebo [SMD + 0.50; 95% CI 0.15, 0.86; I^2^ = 40%; *p* = 0.005 (Figure 7)]. The subgroup meta-analysis of flavanol and coffee polyphenols both showed no significant benefits for skin hydration compared to placebo, probably because of fewer studies. Nonetheless, the pooled meta-analysis of all nine studies (*n* = 495) showed that polyphenols significantly restored the moisture content [SMD + 0.58; 95% CI 0.25, 0.91; I^2^ = 67%; *p* = 0.0006 (Figure 7)]. In these meta-analyses, the studies on procyanidin were moderately heterogeneous (I^2^ = 40%) and the studies on other polyphenols were remarkably heterogeneous, which, to a great extent, was due to insufficient RCTs. Thus, further researches are needed to determine the effects of polyphenols on the moisture content of the skin.

Four of nine studies investigated the influence of polyphenols on skin TEWL. One study explored procyanidin; one study explored flavanol; one study explored coffee polyphenols, and one study explored green tea polyphenols. The RCTs on coffee polyphenols and green tea polyphenols gave a positive effect on decreasing TEWL, while a non-significant effect on reducing TEWL was obtained in studies on procyanidin and flavanol. Thus, further researches are needed to determine the effect of polyphenols on skin TEWL.

Four of the nine studies investigating polyphenols mentioned safety. Two reported no AE, and 1 reported no TAE, and 1 reported AE but no TAE.

### Carotenoids

Carotenoids were tested in seven researches (*n* = 599), three of which focused on single-preparation products (78–80) and four of which highlighted combination preparation products (48, 81–83). The active ingredients involved in the studies included astaxanthin (five studies), zeaxanthin (two studies), and lutein (1 study). Five studies reported the daily dosages of astaxanthin between 2 and 4 mg, which were administered over 6 to 12 weeks. The dosages of zeaxanthin and lutein, which were reported in one study as a combination preparation product were 0.3 mg and 5 mg daily, respectively.

Details in trial designs and methods were commonly absent across the researches, apart from the research of Ito 2018b which was judged to be at low risks of SB, PB, and DB. The risk of AB was low in five researches (0–8.8% attrition). Four of the seven researches were regarded as having high risk of reporting bias for the lack of trial registration and the usage of graphical representations rather than absolute values. Three of the seven researches were at high risk of other biases caused by the potential conflict of interests in two RCTs and the lack of details on life habits and test environment in one RCT.

**Figure 8:**
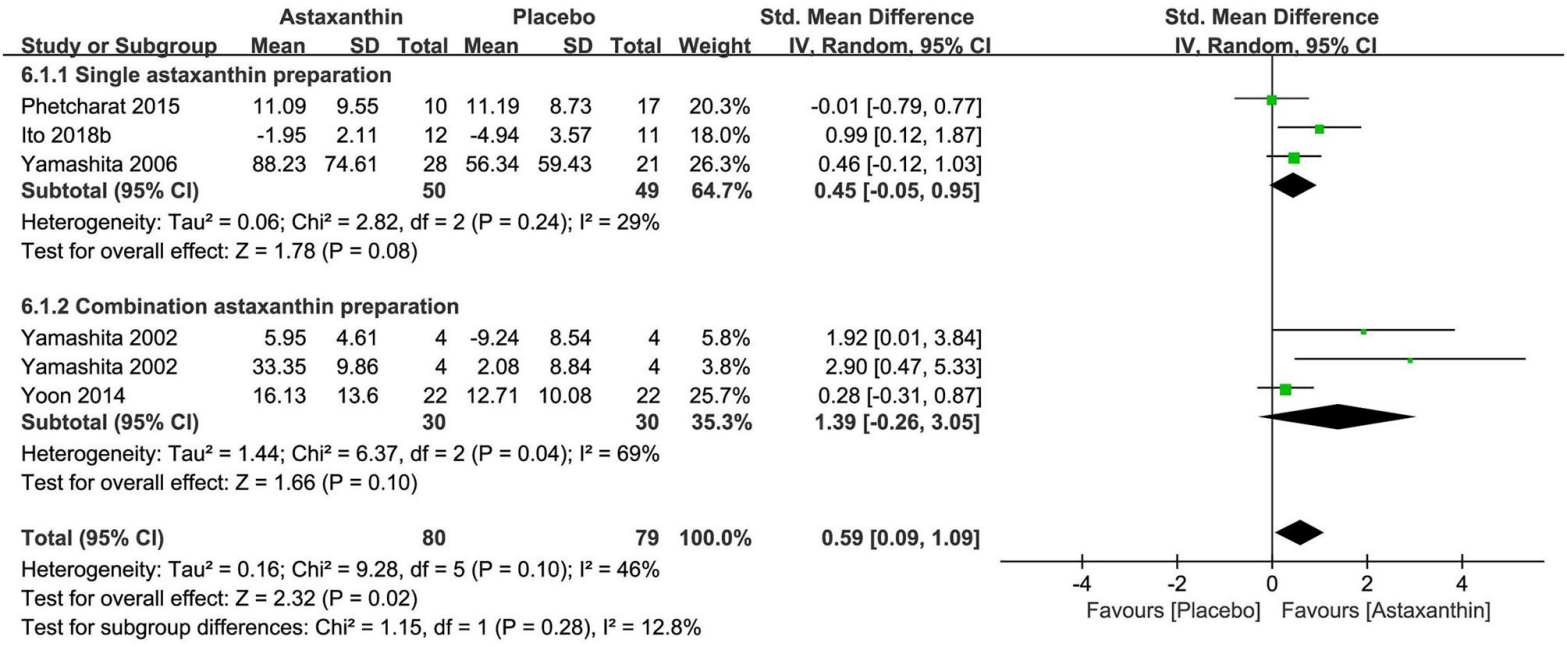
Forest plot of comparison: Astaxanthin vs. placebo on skin hydration (SMD). The details of each study are reported in Supplementary Table S2. CI, confidence interval.

The meta-analysis of three researches (*n* = 99) exploring astaxanthin as a single preparation displayed a non-significant effect on improving stratum corneum water content in contrast to placebo [SMD + 0.45; 95% CI −0.05, 0.95; I^2^ = 29%; *p* = 0.08 (Figure 8)]. Although the meta-analysis of all 5 researches (*n* = 159) exploring astaxanthin showed a significant effect on improving skin hydration in contrast to placebo [SMD + 0.59; 95% CI 0.09, 1.09; I^2^ = 46%; *p* = 0.02 (Figure 8)], the consequence might be resulted from the collagen and tocotrienol in the combination preparation products. None of the five studies explored the effect of astaxanthin on skin TEWL. Zeaxanthin and lutein were excluded from meta-analysis, as no studies investigated them as a single preparation though statistically significant improvements in hydration were observed after the intake of them as combination products in both two studies.

Four of the 7 studies reported safety and no adverse events were found during treatment.

### Aloe

Aloe extract was examined in three studies (84–86) (*n* = 244) as a single-preparation product over 8 to 12 weeks. The daily dosages of aloe vera gel powder were reported in two studies as 0.25 and 0.5 g, respectively. All studies were rated as low risks of SB, PB, DB, and AB. None of the studies was at a high risk of reporting bias or other biases. Of the three researches, only one study revealed a statistically remarkable difference in skin moisture of +8.7 au compared to placebo, and 1 research mentioned a statistically significant difference in TEWL of −1.1g/h/m^2^ in contrast to placebo. Safety was discussed in three trials without treatment-related adverse effects, and one study with adverse events reported similarities between the treatment group and placebo group.

### Turmeric

Turmeric or *Curcuma longa* was explored in two researches (87, 88) (*n* = 75) over 4 to 8 weeks. One study explored the roles of a hot water extract of *C. longa* (WEC) in skin conditions as a single agent and herb preparation in combination with curcumin. Another study compared the effects of turmeric tablets vs. placebo on skin barrier function. Both studies were at a low ROB for the majority of domains with adequate reporting of the trial designs and methods. No studies were at a high risk of other biases. Clinically and statistically, 1 study displayed a remarkable (about +30.5 au) effect on the moisture content in facial skin between +10.7 and +50.3 au compared to placebo. Both studies showed no remarkable diversities in transepidermal water loss between the turmeric group and placebo group. Safety was discussed in both trials and the quantity of AE displayed no difference between the treatment group and placebo group.

### Porcine Placenta Extract

Porcine Placenta Extracts were explored in two researches (89, 90) (*n* = 65), one of which focused on a single-preparation product, and the other highlighted a combination preparation combined with MCT oil, fish oil, olive oil, and other kinds of oils. The daily dosages of Porcine Placenta Extracts were reported in two studies as 0.2 and 0.6 g over 4 to 24 weeks. Both studies were at low risks of SB, AB, and RB, nevertheless, both of them were at high risks of other biases as a consequence of the potential conflict of interests or inadequate reporting on life habits and test conditions. Clinically and statistically, 1 study reported a remarkable increase in hydration on both the cheeks and arms and a decrease in TEWL on the arms. However, another study reported that there were no different changes in skin hydration and TEWL values in the facial skin between the placebo group and test group. Safety data were mentioned in the two researches, with no AE emerging in the test and placebo groups.

## Discussion

The present research selected 66 RCTs regarding dietary supplements for skin moisturizing, involving an overall 4,090 subjects. “Skin hydration or stratum corneum water content” as the first outcome and “transepidermal water loss (TEWL)” as the second outcome was applied to assess the effect of dietary supplements on skin moisturizing. In healthy skin, TEWL is directly proportional to skin hydration. However, the inverse ratios of TEWL and skin moisturization are common in substantial skin illness (91), e,g., elevated TEWL with reduced skin moisturization is observed in atopic dermatitis and keratinization disorder.

### Oral Dietary Supplement Effects on Skin Moisturizing

**Figure 9:**
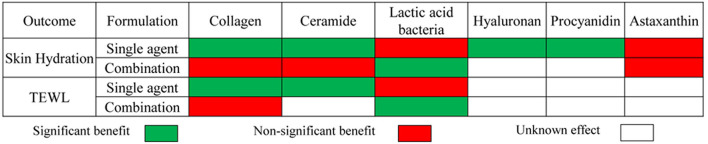
Summary of meta-analysis of studies reporting the effect of dietary supplements on skin moisturizing.

Figure 9 displays the results of meta-analyses regarding the effect of dietary supplements on moisturizing. In most cases, the dietary supplement either produced a significant benefit for improving dryness with decreased TEWL or produced a non-significant effect on moisturizing with unnoticeable changes in TEWL. For example, the meta-analysis displayed that certain dietary supplements exert a statistically remarkable effect on increasing skin hydration and reducing the values of TEWL compared to placebo, including collagen as a single agent, ceramides as a single agent, and lactic acid bacteria as a combination preparation. Meanwhile, no statistically significant effects on both skin hydration and TEWL were observed for collagen as a combination preparation and lactic acid bacteria as a single agent.

Most dietary supplements as combination preparation had exactly the opposite effect on moisturizing in contrast to those as a single agent. We prefer the latter to the former for reasons as follows. First, there was a lower statistical heterogeneity in the studies investigating the supplements, as a single agent, e.g., owing to the lack of statistical inhomogeneity (I^2^ = 0, Figure 3), it's more dependable to consider that within the 2 types of treatment, the oral collagen supplement, as a single agent, could significantly increase skin hydration, compared with the control group. Second, insufficient studies investigating dietary supplements as combination preparations were included in the research on moisturizing, especially, since there were only 2 RCTs investigating ceramide as a combination preparation. Finally, it is unreasonable to ignore the potential false positive results in studies investigating combination dietary supplements containing ingredients with positive effects on skin care or the potential false negative results in studies investigating combination dietary supplements containing ingredients with negative effects on skin protection. For instance, in studies investigating lactic acid bacteria or astaxanthin as a combination preparation, there were many other ingredients with positive effects on skin care, such as honeybush extract, galacto-oligosaccharides, or collagen, which might cause false positive results to some extent. Therefore, in order to assess a specific dietary supplement, its application effect, as combination preparation, on skin health was a less useful guide for skin moisture assessment, and will not be taken into account in the following analysis.

In view of the aforesaid results, the meta-analyses demonstrated that both collagen and ceramides exhibited statistically significant effects on improving skin hydration and causing lower values of TEWL. Lactic acid bacteria or lactobacillus drink displayed a non-significant benefit for skin moisturizing as per the outcomes herein. Oral hyaluronan was also beneficial for improving skin dryness, whose effects on TEWL need further research because of insufficient RCTs. As for polyphenols, by subgroup analysis, no statistically significant effects on moisturizing dry skin were seen, apart from procyanidin which had an unclear impact on TEWL owing to inadequate RCTs. The conclusion might be remarkably different according to the gathered outcomes of meta-analysis regarding skin water content if more studies researching polyphenols were included in the subgroup meta-analysis. The meta-analysis on the roles of astaxanthin in skin hydration, however, displayed a significantly different result compared with the study of Zhou 2021 (92) which neglected the potential false positive result. In terms of presenting data, by ruling out the possible influence of other ingredients with positive effects, we tended to think that astaxanthin had a non-significant effect on skin hydration according to the result of the subgroup meta-analysis, which was consistent with the conclusion from the study of Tominaga 2012 (93).

Statistically and significantly improved skin conditions were observed for other dietary supplements such as Aloe compared to placebo in a few articles, but each of these was explored in 3 or even 2 researches. Therefore, meta-analysis made no sense for these supplements. Therefore, there is an urgent need for longer RCTs with bigger sample sizes and the usage of objective dermatologic methods to obtain more dependable outcomes pertaining to the effects of dietary supplements on skin health.

Safety data were mentioned briefly in most research on RCTs underpinning the present paper. Based on the insufficient available data, we can conclude that the application of dietary supplements in skin care is basically safe as no treatment-related adverse effects proposed in reports were included in this review.

### Mechanism

Although some of these dietary supplements have long been one of the most popular supplements for skin moisturizing, the precise potential causal link related to the avoidance of skin dryness by these supplements remained elusive.

The stratum corneum, as the outermost tier of the epidermis, protects the organism from water loss, serving as a thin physical, chemical, and immunological barrier. It is of great importance to maintain epidermal physiologic homeostasis such as a proper moisture level for the normal proliferation and differentiation of the epidermis. Epidermal water-holding capabilities tightly regulate the water content in or on the skin and they depend on three major components (2, 94): one is normal keratinization as well as a natural moisturizing factor (NMF) within corneocytes; the other is the orderly arrangement of the SC intercellular lipids to prevent transepidermal water loss (TEWL) via corneocyte adhesion to form this barrier. At last, it has been found that the water-transporting protein AQP3 in the viable epidermis and tight junction architectures at the junction between the stratum granulosum and SC is related to skin barrier function via water transportation in the skin.

The oral intake of collagen peptides has been found to increase the moisturizing capacity of the skin by enhancing the moisture content of the stratum corneum. Collagen is well absorbed into the body in the form of amino acids (AA), dipeptides, and tripeptides after ingestion via the oral route (95). Some research works have displayed the remarkable absorption of two main CPs, i.e., prolyl-hydroxyproline (Pro–Hyp) and hydroxyprolyl-glycine (Hyp–Gly) that exert chemotaxis on dermal fibroblasts and reinforce cellular proliferative ability (27). In addition, Pro-Hyp reinforces the generation of hyaluronic acid which has been discovered to be pivotal for keratinocyte proliferation and differentiation (96) by up-regulating hyaluronic synthase 2 (HAS2) mRNA contents (25). Moreover, orally administered CP was found to promote filaggrin expression (97) which is involved in the maintenance of the internal structure of keratinocytes, the increase of the level of NMF constituents, and the repair of skin barrier functions to improve TEWL.

Ceramides (CERs), as vital constituents of the intercellular lipids of the SC, are vital for establishing and sustaining the water-preserving performances of the skin. The sphingoid base architectures in SC CERs are produced by the decomposition of extracutaneous lipids deriving from the general diet and can't be synthetized *de novo* by ourselves just like indispensable fatty acids, sphingosines, and sphinganines (39). Orally administered plant glucosylceramides (GlcCERs) derived from rice and konjac can elevate epidermis CERs by mechanisms including direct localization and usage of absorbed diet CERs in the epidermis without any metabolism conversion, the usage of exogenetic GlcCERs metabolins by keratinocytes to establish their own sphingolipids or the generation of skin CERs by metabolins (98). Other proposed mechanisms regarding skin barrier improvement include the stimulation of collagen synthesis (40), the inhibition of matrix proteases (40), and the elevation of the expression of aquaporin-3 (46). The improvement in skin moisture content resulting from the oral intake of ceramides is likely to be induced by the combination of the mechanisms described above.

Although meta-analysis on the effect of lactic acid bacteria on skin moisturizing showed a non-significant result compared to placebo, a small number of studies indicated a slight improvement in skin barrier function after the daily intake of the lactic acid bacteria beverage. Phenols, like phenol and p-cresol, as the metabolins of aromatic amino acids generated by intestinal microbes, are considered biologically active toxins and serum markers of gut milieu disturbance (99). It has been revealed that phenols are capable of disturbing the differentiative activity of monolayer-cultivated keratinocytes *in vitro* and that phenols generated by intestinal microbes accumulate in the skin through the circulation and are capable of disturbing the differentiative activity of keratinocytes in hairless mice (100). As a tool to modulate gut microbiota, probiotics are anticipated to assist in sustaining a healthy skin via the reduction of phenols generation via intestinal microflora (101). Nonetheless, it may not act rapidly and directly on the stratum corneum and may have an obvious effect only on people with intestinal flora disturbance, compared to collagen and ceramide.

As essential components of skin, hyaluronan plays an important role in water absorption and decides skin water contents. After the ingestion of hyaluronan, both the partly depolymerized and the completely intact HA are absorbed and distributed to the skin, which increases HA production in human fibroblasts and promotes cellular proliferative ability in the production of mankind fibroblast populated collagen lattices (102). As a result, this elevation of the cellular quantity inhibits the skin's moisture loss, which contributes to moisturizing the skin.

Polyphenols and carotenoids, as the best-known antioxidants, have been widely applied in whitening and wrinkle defense. Procyanidin, belonging to polyphenols, has shown a potential benefit for reducing dry skin based on the relevant meta-analysis. An increase in the expression of aquaporin 3 (AQP3) can be attributed to the mechanism pertaining to the improvement of the moisture contents of the stratum corneum by procyanidin which plays an important role in maintaining skin moisturization (103). Astaxanthin, as a member of carotenoids, exerts a positive effect on diluting the melanin and removing oxygen-derived free radicals (104), despite the non-significant result of meta-analysis on skin moisturizing.

In summary, the detailed mechanisms of oral skin moisturizers are complex, however, the aforementioned supplements concerning moisture retention of skin share a common characteristic that they directly or indirectly supplement the nutrients for healthy skin so as to repair the skin barrier and function, achieving the improvement of skin hydration. Therefore, even though many foods such as fruits and vegetables can replenish the body's water, they cannot fundamentally restore the water storage capacity of the stratum corneum, resulting in persistent dry skin.

## Conclusion

Holistically, many of the dietary supplements produced a statistically significant effect on skin moisturizing compared to placebo, whereas there were still substantial skin-friendly supplements presenting no obvious beneficial effects on moisturizing. Some dietary supplements warrant more exploration via larger and more stringent research to identify the effect size, especially when it comes to polyphenols, carotenoids, aloe, and certain supplements merely reported by one research. Massive selected researches were small and lacked satisfactory quality in terms of designs and methods. Future studies have to ensure that trials are performed and reported using methods harboring as little bias as possible and in accordance with the CONSORT Statement for the report of clinic trials (105). At present, merely oral dietary supplements including collagen, ceramides, hyaluronan, and procyanidin are proven to be effective in skin moisturizing, whereas for skin moisturizing, the evidence supporting the recommendation of other dietary supplements, such as lactic acid bacteria and astaxanthin, is still insufficient.

## Data Availability Statement

The original contributions presented in the study are included in the article/Supplementary Material, further inquiries can be directed to the corresponding author.

## Author Contributions

QS and JW were involved in the conception and design. QS, GQ, and JW were involved in literature retrieval, data collection, and extraction and analysis. JW and HC were involved in systematic review and meta-analysis and responsible for the final approval of the version to be published. All authors revised and approved the final version of the manuscript.

## Funding

This work was financially supported by Sichuan Key Technologies Research and Development Program (Nos. 2022YFS0416 and 2022YFS0413).

## Conflict of Interest

GQ was employed by Haisco Pharmaceutical Group Company Ltd. The remaining authors declare that the research was conducted in the absence of any commercial or financial relationships that could be construed as a potential conflict of interest.

## Publisher's Note

All claims expressed in this article are solely those of the authors and do not necessarily represent those of their affiliated organizations, or those of the publisher, the editors and the reviewers. Any product that may be evaluated in this article, or claim that may be made by its manufacturer, is not guaranteed or endorsed by the publisher.

## References

[1] Swann G. The skin is the body's largest organ. J Vis Commun Med. (2010) 33:148–9. 10.3109/17453054.2010.525439 21087182

[2] Verdier-Sévrain S Bonté F. Skin hydration: a review on its molecular mechanisms. J Cosmet Dermatol. (2007) 6:75–82. 10.1111/j.1473-2165.2007.00300.x 17524122

[3] Son ED Kim Y Joo KM Kim HJ Lee E Nam GW . Skin dryness in apparently healthy human skin is associated with decreased expression of bleomycin hydrolase in the stratum corneum. Clin Exp Dermatol. (2015) 40:247–53. 10.1111/ced.12520 25495994

[4] Andriessen A. Prevention, recognition and treatment of dry skin conditions. Br J Nurs. (2013) 22:26–30. 10.12968/bjon.2013.22.1.26 23299208

[5] Draelos ZD. The science behind skin care: moisturizers. J Cosmet Dermatol. (2018) 17:138–44. 10.1111/jocd.12490 29319217

[6] Lodén M. The clinical benefit of moisturizers. JEADV. (2005) 19:672–88. 10.1111/j.1468-3083.2005.01326.x 16268870

[7] Lodén M. Role of topical emollients and moisturizers in the treatment of dry skin barrier disorders. Am J Clin Dermatol. (2003) 4:771–88. 10.2165/00128071-200304110-00005 14572299

[8] Sethi A Kaur T Malhotra SK Gambhir ML. Moisturizers: the slippery road. Indian J Dermatol. (2016) 61:279–87. 10.4103/0019-5154.182427 27293248 PMC4885180

[9] Lodén M. Do moisturizers work? J Cosmet Dermatol. (2003) 2:141–9. 10.1111/j.1473-2130.2004.00062.x 17163920

[10] Jacobs DR. Jr. Tapsell LC. Food, not nutrients, is the fundamental unit in nutrition Nutr Rev. (2007) 65:439–50. 10.1111/j.1753-4887.2007.tb00269.x 17972438

[11] Faria-Silva C Ascenso A Costa AM Marto J Carvalheiro M Ribeiro HM . Feeding the skin: a new trend in food and cosmetics convergence. Trends Food Sci Technol. (2020) 95:21–32. 10.1016/j.tifs.2019.11.015

[12] Vollmer DL West VA Lephart ED. Enhancing skin health: by oral administration of natural compounds and minerals with implications to the dermal microbiome. Int J Mol Sci. (2018) 19:3059. 10.3390/ijms19103059 30301271 PMC6213755

[13] Chang J. Medicinal herbs: drugs or dietary supplements? Biochem Pharmacol. (2000) 59:211–9. 10.1016/s0006-2952(99)00243-9 10609549

[14] Ramos-e-Silva M Celem LR Ramos-e-Silva S Fucci-da-Costa AP. Anti-Aging cosmetics: facts and controversies. Clin Dermatol. (2013) 31:750–58. 10.1016/j.clindermatol.2013.05.013 24160281

[15] Moher D Liberati A Tetzlaff J Altman DG. Preferred reporting items for systematic reviews and meta-analyses: the prisma statement. Int J Surg. (2010) 8:336–41. 10.1016/j.ijsu.2010.02.007 20171303

[16] Digitizer GG. Getdata-Graph-Digitizer 2.26. Available online at: Http://Getdata-Graph-Digitizer.Com/ (accessed on 19 December 2021).

[17] Higgins JPT Altman DG Gøtzsche PC Jüni P Moher D Oxman AD . The cochrane collaboration's tool for assessing risk of bias in randomised trials. BMJ. (2011) 343:d5928. 10.1136/bmj.d5928%JBMJ. 22008217 PMC3196245

[18] The The Nordic Cochrane Centre T.C.C. Review Manager (Revman). (2020). Available online at: Https://Training.Cochrane.Org/Onlinelearning/Core-Software-Cochrane-Reviews/Revman (accessed on 19 December 2021).

[19] Higgins Jpt Thomas J Chandler J Cumpston M Li T Page Mj Welch Va (editors). Cochrane Handbook for Systematic Reviews of Interventions Version 6.3. (updated February 2022) Cochrane. (2022). Available online at: www.Training.Cochrane.Org/Handbook.

[20] Ursoniu S Sahebkar A Serban MC Banach M. Lipid profile and glucose changes after supplementation with astaxanthin: a systematic review and meta-analysis of randomized controlled trials. AMS. (2015) 11:253–66. 10.5114/aoms.2015.50960 25995739 PMC4424245

[21] Borenstein M Hedges L Higgins J Rothstein H. Identifying and Quantifying Heterogeneity. Introduction to meta-analysis. Chichester, UK: John Wiley & Sons (2021). p. 99–117.

[22] Higgins JPT Thompson SG Deeks J Altman DG. Measuring inconsistency in meta-analysis. Br Med J. (2003) 327:557–60. 10.1136/bmj.327.7414.557 12958120 PMC192859

[23] Asserin J Lati E Shioya T Prawitt J. The effect of oral collagen peptide supplementation on skin moisture and the dermal collagen network: evidence from an *ex vivo* model and randomized, placebo-controlled clinical trials. J Cosmet Dermatol. (2015) 14:291–301. 10.1111/jocd.12174 26362110

[24] Choi SY Ko EJ Lee YH Kim BG Shin HJ Seo DB . Effects of collagen tripeptide supplement on skin properties: a prospective, randomized, controlled Study. J Cosmet Laser Ther. (2014) 16:132–7. 10.3109/14764172.2013.854119 24131075

[25] Inoue N Sugihara F Wang X. Ingestion of bioactive collagen hydrolysates enhance facial skin moisture and elasticity and reduce facial ageing signs in a randomised double-blind placebo-controlled clinical study. J Sci Food Agric. (2016) 96:4077–81. 10.1002/jsfa.7606 26840887

[26] Kim DU Chung HC Choi J Sakai Y Lee BY. Oral intake of low-molecular-weight collagen peptide improves hydration, elasticity, and wrinkling in human skin: a randomized, double-blind, placebo-controlled study. Nutrients. (2018) 10:826. 10.3390/nu10070826 29949889 PMC6073484

[27] Koizumi S Inoue N Shimizu M Kwon C-j Kim H-y Park K. Effects of dietary supplementation with fish scales-derived collagen peptides on skin parameters and condition: a randomized, placebo-controlled, double-blind study. Int J Peptide Res Therapeutics. (2017) 24:397–402. 10.1007/s10989-017-9626-0

[28] Koizumi S Sugihara F Inoue N Wang X. The effects of collagen hydrolysates derived from tilapia scales or skin on human facial skin-a randomized double-blind placebo-controlled clinical study. JPT. (2019) 47:57–63.

[29] Maia Campos P Melo MO Siqueira César FC. Topical application and oral supplementation of peptides in the improvement of skin viscoelasticity and density. J Cosmet Dermatol. (2019) 18:1693–9. 10.1111/jocd.12893 30834689

[30] Miyanaga M Uchiyama T Motoyama A Ochiai N Ueda O Ogo M. Oral supplementation of collagen peptides improves skin hydration by increasing the natural moisturizing factor content in the stratum corneum: a randomized, double-blind, placebo-controlled clinical trial. Skin Pharmacol Physiol. (2021) 34:115–27. 10.1159/000513988 33774639

[31] Sugihara F Inoue N Wang X. Clinical effects of ingesting collagen hydrolysate on facial skin properties: -a randomized, placebo-controlled, double-blind trial. JPT. (2015) 43:67–70.

[32] Tak YJ Shin DK Kim AH Kim JI Lee YL Ko HC . Effect of collagen tripeptide and adjusting for climate change on skin hydration in middle-aged women: a randomized, double-blind, placebo-controlled trial. Front Med. (2021) 7:608903. 10.3389/fmed.2020.608903 33521019 PMC7839319

[33] Bolke L Schlippe G Gerß J Voss W. A Collagen supplement improves skin hydration, elasticity, roughness, and density: results of a randomized, placebo-controlled, blind study. Nutrients. (2019) 11:2494. 10.3390/nu11102494 31627309 PMC6835901

[34] Ito N Seki S Ueda F. Effects of composite supplement containing collagen peptide and ornithine on skin conditions and plasma Igf-1 levels-a randomized, double-blind, placebo-controlled trial. Marine Drugs. (2018) 16:482. 10.3390/md16120482 30513923 PMC6315531

[35] Lin P Alexander RA Liang CH Liu C Lin YH Lin YH . Collagen formula with djulis for improvement of skin hydration, brightness, texture, crow's feet, and collagen content: a double-blind, randomized, placebo-controlled trial. J Cosmet Dermatol. (2021) 20:188–94. 10.1111/jocd.13500 32530124

[36] Primavera G Berardesca E. Clinical and instrumental evaluation of a food supplement in improving skin hydration. Int J Cosmet Sci. (2005) 27:199–204. 10.1111/j.1467-2494.2005.00237.x 18492188

[37] Schwartz SR Hammon KA Gafner A Dahl A Guttman N Fong M . Novel hydrolyzed chicken sternal cartilage extract improves facial epidermis and connective tissue in healthy adult females: a randomized, double-blind, placebo-controlled trial. Altern Ther Health Med. (2019) 25:12–29. 31221944

[38] Žmitek K Žmitek J Rogl Butina M Pogačnik T. Effects of a combination of water-soluble coenzymeq10 and collagen on skin parameters and condition:results of a randomised, placebo-controlled,double-blind study. Nutrients. (2020) 12:618. 10.3390/nu12030618 32120787 PMC7146335

[39] Bizot V Cestone E Michelotti A Nobile V. Improving skin hydration and age-related symptoms by oral administration of wheat glucosylceramides and digalactosyl diglycerides: a human clinical study. Cosmetics. (2017) 4:37. 10.3390/cosmetics4040037

[40] Boisnic S Keophiphath M Serandour AL Branchet MC Le Breton S Lamour I . Polar lipids from wheat extract oil improve skin damages induced by aging: evidence from a randomized, placebo-controlled clinical trial in women and an *ex vivo* study on human skin explant. J Cosmet Dermatol. (2019) 18:2027–36. 10.1111/jocd.12967 31033133

[41] Guillou S Ghabri S Jannot C Gaillard E Lamour I Boisnic S. The moisturizing effect of a wheat extract food supplement on women's skin: a randomized, double-blind placebo-controlled trial. Int J Cosmet Sci. (2011) 33:138–43. 10.1111/j.1468-2494.2010.00600.x 20646083

[42] Hori M Kishimoto S Tezuka Y Nishigori H Nomoto K Hamada U . Double-Blind study on effects of glucosyl ceramide in beet extract on skin elasticity and fibronectin production in human dermal fibroblasts. Anti-Aging Medicine. (2010) 7:129–42. 10.3793/jaam.7.129

[43] Nishidc A. Shimura M. Ohnuki K. Shimizu K. Ohnukr K. Effects of oral use of phytosphingosine on skin moisturizing in healthy adults-a 12-week double-blind, randomized, placebo-controlled trial Japanese pharmacology and therapeutics. (2020) 48:237–41.

[44] Oda T Tachimoto H Kishi M Kaga T Ichihashi M. Effect of oral intake of ceramide-containing acetic acid bcteria on skin barrier function. Anti-Aging Med. (2010) 7:50–4. 10.3793/jaam.7.5033658470

[45] Takara T Yamamoto K Suzuki N Yamashita S Iio S-i Noguchi H . Oryza ceramide^®^, a rice-derived extract consisting of glucosylceramides and ?-sitosterol glucoside, improves facial skin dehydration in Japanese subjects. Funct Foods Health Dis. (2021) 11:385. 10.31989/ffhd.v11i8.809

[46] Tsuchiya Y Ban M Kishi M Ono T. Safety evaluation of the excessive intake of ceramide-containing acetic acid bacteria - a randomized, double-blind, placebo-controlled study over a 4-week period. J Oleo Sci. (2020) 69:1497–508. 10.5650/jos.ess20198 33658470

[47] Uchiyama T Nakano Y Ueda O Mori H Nakashima M Noda A . Oral intake of glucosylceramide improves relatively higher level of transepidermal water loss in mice and healthy human subjects. J Health Sci. (2008) 54:559–66. 10.1248/jhs.54.559

[48] Schwartz S Frank E Gierhart D Simpson P Frumento R. Zeaxanthin-Based dietary supplement and topical serum improve hydration and reduce wrinkle count in female subjects. J Cosmet Dermatol. (2016) 15:e13–20. 10.1111/jocd.12226 27312122

[49] Chan LP Tseng YP Liu C Liang CH. Fermented pomegranate extracts protect against oxidative stress and aging of skin. J Cosmet Dermatol. (2021). 10.1111/jocd.14379 34416060

[50] Chan L-P Tseng Y-P Liu C Liang C-H. Anti-Oxidant and anti-aging activities of fermented vegetable-fruit drink. J Food Nutr Res. (2021) 9:240–50. 10.12691/jfnr-9-5-1

[51] Gueniche A Philippe D Bastien P Reuteler G Blum S Castiel-Higounenc I . Randomised double-blind placebo-controlled study of the effect of lactobacillus paracasei Ncc 2461 on skin reactivity. Benef Microbes. (2014) 5:137–45. 10.3920/bm2013.0001 24322879

[52] Kim H Kim HR Jeong BJ Lee SS Kim TR Jeong JH . Effects of oral intake of kimchi-derived lactobacillus plantarum K8 lysates on skin moisturizing. J Microbiol Biotechnol. (2015) 25:74–80. 10.4014/jmb.1407.07078 25179904

[53] Kimoto-Nira H Moriya N Sasaki K Suzuki C. Effects of ingesting milk fermented by lactococcus lactis H61 on skin properties and health biomarkers in middle-aged women: a randomized, double-blind study. J Aging Res Clin Pract. (2015) 4:109–15. 10.14283/jarcp.2015.57

[54] Kimoto-Nira H Nagakura Y Kodama C Shimizu T Okuta M Sasaki K . Effects of Ingesting milk fermented by lactococcus lactis H61 on skin health in young women: a randomized double-blind study. J Dairy Sci. (2014) 97:5898–903. 10.3168/jds.2014-7980 25022690

[55] Lee DE Huh CS Ra J Choi ID Jeong JW Kim SH . Clinical evidence of effects of lactobacillus plantarum Hy7714 on skin aging: a randomized, double blind, placebo-controlled study. J Microbiol Biotechnol. (2015) 25:2160–8. 10.4014/jmb.1509.09021 26428734

[56] Nagino T Kaga C Kano M Masuoka N Anbe M Moriyama K . Effects of fermented soymilk with lactobacillus casei shirota on skin condition and the gut microbiota: a randomised clinical pilot trial. Benef Microbes. (2018) 9:209–18. 10.3920/bm2017.0091 29264969

[57] Ogawa M Saiki A Matsui Y Tsuchimoto N Nakakita Y Takata Y . Effects of oral intake of heat-killed lactobacillus brevis Sbc8803 (Sbl88™) on dry skin conditions: a randomized, double-blind, placebo-controlled study. Exp Ther Med. (2016) 12:3863–72. 10.3892/etm.2016.3862 28105118 PMC5228549

[58] Saito Y Mihara T Maruyama K Saito J Ikeda M Tomonaga A . Effects of intake of lactobacillus casei subsp. Casei 327 on skin conditions: a randomized, double-blind, placebo-controlled, parallel-group study in women. Biosci Microbiota Food Health. (2017) 36:111–20. 10.12938/bmfh.16-031 28748132 PMC5510156

[59] Choi SY Hong JY Ko EJ Kim BJ Hong SW Lim MH . Protective effects of fermented honeybush (Cyclopia Intermedia) extract (Hu-018) against skin aging: a randomized, double-blinded, placebo-controlled study. J Cosmet Laser Ther. (2018) 20:313–8. 10.1080/14764172.2017.1418512 29388846

[60] Kano M Masuoka N Kaga C Sugimoto S Iizuka R Manabe K . Consecutive intake of fermented milk containing bifidobacterium breve strain yakult and galacto-oligosaccharides benefits skin condition in healthy adult women. Biosci Microbiota Food Health. (2013) 32:33–9. 10.12938/bmfh.32.33 24936360 PMC4034291

[61] Mori N Kano M Masuoka N Konno T Suzuki Y Miyazaki K . Effect of probiotic and prebiotic fermented milk on skin and intestinal conditions in healthy young female students. Biosci Microbiota Food Health. (2016) 35:105–12. 10.12938/bmfh.2015-022 27508111 PMC4965514

[62] Puch F Samson-Villeger S Guyonnet D Blachon JL Rawlings AV Lassel T. Consumption of functional fermented milk containing borage oil, green tea and vitamin E enhances skin barrier function. Exp Dermatol. (2008) 17:668–74. 10.1111/j.1600-0625.2007.00688.x 18318715

[63] Hong YH Chang UJ Kim YS Jung EY Suh HJ. Dietary galacto-oligosaccharides improve skin health: a randomized double blind clinical trial. Asia Pac J Clin Nutr. (2017) 26:613–8. 10.6133/apjcn.052016.05 28582809

[64] W GGSF. Stability and Clinical Efficacy of Honeybush Extracts in Cosmeceutical Product (dissertation/master's thesis). Potchefstroom, North-West University (2012).

[65] Hsu TF Su ZR Hsieh YH Wang MF Oe M Matsuoka R . Oral hyaluronan relieves wrinkles and improves dry skin: a 12-week double-blinded, placebo-controlled study. Nutrients. (2021) 13:2220. 10.3390/nu13072220 34203487 PMC8308347

[66] Kawada C Yoshida T Yoshida H Sakamoto W Odanaka W Sato T . Ingestion of hyaluronans (molecular weights 800 K and 300 K) improves dry skin conditions: a randomized, double blind, controlled study. J Clin Biochem Nutr. (2015) 56:66–73. 10.3164/jcbn.14-81 25834304 PMC4306664

[67] Michelotti A Cestone E De Ponti I Pisati M Sparta E Tursi F. Oral intake of a new full-spectrum hyaluronan improves skin profilometry and ageing: a randomized, double-blind, placebo-controlled clinical trial. EJD. (2021) 31:798–805. 10.1684/ejd.2021.4176 34933842

[68] Sato T Sakakoto W Odanaka W Yoshida K Urishibata O. Clinical effects of dietary hyaluronic acid on dry, rough skin. Aesthetic Dermatology. (2002) 12:109–20.

[69] Buonocore D Lazzeretti A Tocabens P Nobile V Cestone E Santin G . Resveratrol-Procyanidin blend: nutraceutical and antiaging efficacy evaluated in a placebocontrolled, double-blind study. Clin Cosmet Investig Dermatol. (2012) 5:159–65. 10.2147/ccid.S36102 23071399 PMC3469214

[70] Hughes-Formella B Wunderlich O Williams R. Anti-Inflammatory and skin-hydrating properties of a dietary supplement and topical formulations containing oligomeric proanthocyanidins. Skin Pharmacol Physiol. (2007) 20:43–9. 10.1159/000096171 17035721

[71] Shoji T Masumoto S Moriichi N Ohtake Y Kanda T. Administration of apple polyphenol supplements for skin conditions in healthy women: a randomized, double-blind, placebo-controlled clinical trial. Nutrients. (2020) 12(4). 10.3390/nu12041071 32294883 PMC7231294

[72] Tsuchiya T Fukui Y Izumi R Numano K Zeida M. Effects of oligomeric proanthocyanidins (opcs) of red wine to improve skin whitening and moisturizing in healthy women - a placebo-controlled randomized double-blind parallel group comparative study. Eur Rev Med Pharmacol Sci. (2020) 24:1571–84. 10.26355/eurrev_202002_20215 32096209

[73] Heinrich U Neukam K Tronnier H Sies H Stahl W. Long-Term ingestion of high flavanol cocoa provides photoprotection against uv-induced erythema and improves skin condition in women. J Nutr. (2006) 136:1565–9. 10.1093/jn/136.6.1565 16702322

[74] Mogollon JA Boivin C Lemieux S Blanchet C Claveau J Dodin S. Chocolate flavanols and skin photoprotection: a parallel, double-blind, randomized clinical trial. Nutr J. (2014) 13:66. 10.1186/1475-2891-13-66 24970388 PMC4082621

[75] Fukagawa S Haramizu S Sasaoka S Yasuda Y Tsujimura H Murase T. Coffee polyphenols extracted from green coffee beans improve skin properties and microcirculatory function. Biosci Biotechnol Biochem. (2017) 81:1814–22. 10.1080/09168451.2017.1345614 28675091

[76] Tseng YP Liu C Chan LP Liang CH. Coffee pulp supplement affects antioxidant status and favors anti-aging of skin in healthy subjects. J Cosmet Dermatol. (2021). 10.1111/jocd.14341 34265165

[77] Heinrich U Moore CE De Spirt S Tronnier H Stahl W. Green tea polyphenols provide photoprotection, increase microcirculation, and modulate skin properties of women. J Nutr. (2011) 141:1202–8. 10.3945/jn.110.136465 21525260

[78] Ito N Seki S Ueda F. The protective role of astaxanthin for uv-induced skin deterioration in healthy people-a randomized, double-blind, placebo-controlled trial. Nutrients. (2018) 10:817. 10.3390/nu10070817 29941810 PMC6073124

[79] Phetcharat L Wongsuphasawat K Winther K. The effectiveness of a standardized rose hip powder, containing seeds and shells of rosa canina, on cell longevity, skin wrinkles, moisture, and elasticity. Clin Interv Aging. (2015) 10:1849–56. 10.2147/cia.S90092 26604725 PMC4655903

[80] Yamashita E. The effects of a dietary supplement containing astaxanthin on skin condition. Food Style. (2006) 9:72.

[81] Yamashita E. Cosmetic benefit of dietary supplements including astaxanthin and tocotrienol on human skin. Food Style. (2002) 21:6.

[82] Palombo P Fabrizi G Ruocco V Ruocco E Fluhr J Roberts R . Beneficial long-term effects of combined oral/topical antioxidant treatment with the carotenoids lutein and zeaxanthin on human skin: a double-blind, placebo-controlled study. Skin Pharmacol Physiol. (2007) 20:199–210. 10.1159/000101807 17446716

[83] Yoon HS Cho HH Cho S Lee SR Shin MH Chung JH. Supplementating with dietary astaxanthin combined with collagen hydrolysate improves facial elasticity and decreases matrix metalloproteinase-1 and−12 expression: a comparative study with placebo. J Med Food. (2014) 17:810–6. 10.1089/jmf.2013.3060 24955642

[84] Kaminaka C Yamamoto Y Sakata M Hamamoto C Misawa E Nabeshima K . Effects of low-dose aloe sterol supplementation on skin moisture, collagen score and objective or subjective symptoms: 12-week, double-blind, randomized controlled trial. J Dermatol. (2020) 47:998–1006. 10.1111/1346-8138.15428 32515040 PMC7496846

[85] Tanaka M Misawa E Yamauchi K Abe F Ishizaki C. Effects of plant sterols derived from aloe vera gel on human dermal fibroblasts *in vitro* and on skin condition in japanese women. Clin Cosmet Investig Dermatol. (2015) 8:95–104. 10.2147/ccid.S75441 25759593 PMC4345938

[86] Tanaka M Yamamoto Y Misawa E Nabeshima K Saito M Yamauchi K . Effects of aloe sterol supplementation on skin elasticity, hydration, and collagen score: a 12-week double-blind, randomized, controlled trial. Skin Pharmacol Physiol. (2016) 29:309–17. 10.1159/000454718 28088806

[87] Asada K Ohara T Muroyama K Yamamoto Y Murosaki S. Effects of hot water extract of curcuma longa on human epidermal keratinocytes in vitro and skin conditions in healthy participants: a randomized, double-blind, placebo-controlled trial. J Cosmet Dermatol. (2019) 18:1866–74. 10.1111/jocd.12890 30809971

[88] Vaughn AR Clark AK Notay M Sivamani RK. Randomized controlled pilot study of dietary supplementation with turmeric or herbal combination tablets on skin barrier function in healthy subjects. J Med Food. (2018) 21:1260–5. 10.1089/jmf.2018.001 30457892

[89] Kim K Sung J Lee H Ono T Yonei Y. Effect of a dietary supplement containing porcine placenta extract on skin hydration: a placebo-controlled, randomized, double-blind, clinical study. Japanese Pharmacology Therapeutics. (2018) 46:1023–34.

[90] Nagae M Nagata M Teramoto M Yamakawa M Matsuki T Ohnuki K . Effect of porcine placenta extract supplement on skin condition in healthy adult women: a randomized, double-blind placebo-controlled study. Nutrients. (2020) 12:1671. 10.3390/nu12061671 32512710 PMC7353038

[91] Buraczewska I Berne B Lindberg M Törmä H Lodén M. Changes in skin barrier function following long-term treatment with moisturizers, a randomized controlled trial. Br J Dermatol. (2007) 156:492–8. 10.1111/j.1365-2133.2006.07685.x 17300239

[92] Zhou X Cao Q Orfila C Zhao J Zhang L. Systematic review and meta-analysis on the effects of astaxanthin on human skin ageing. Nutrients. (2021) 13:2917. 10.3390/nu13092917 34578794 PMC8472736

[93] Tominaga K Hongo N Karato M Yamashita E. Cosmetic benefits of astaxanthin on humans subjects. Acta Biochim Pol. (2012) 59:43–7. 22428137

[94] Hashizume H. Skin aging and dry skin. J Dermatol. (2004) 31:603–9. 10.1111/j.1346-8138.2004.tb00565.x 15492432

[95] Maeda K. Skin-moisturizing effect of collagen peptides taking orally. J Nutr Food Sci. (2018) 8:682. 10.4172/2155-9600.1000682

[96] Price RD Berry MG Navsaria HA. Hyaluronic acid: the scientific and clinical evidence. JPRAS. (2007) 60:1110–9. 10.1016/j.bjps.2007.03.005 17466613

[97] Kang MC Yumnam S Kim SY. Oral intake of collagen peptide attenuates ultraviolet B irradiation-induced skin dehydration *in vivo* by regulating hyaluronic acid synthesis. Int J Mol Sci. (2018) 19:3551. 10.3390/ijms19113551 30423867 PMC6274925

[98] Tessema EN Gebre-Mariam T Neubert RHH Wohlrab J. Potential applications of phyto-derived ceramides in improving epidermal barrier function. Skin Pharmacol Physiol. (2017) 30:115–38. 10.1159/000464337 28407621

[99] Miyazaki K Masuoka N Kano M Iizuka R. Bifidobacterium fermented milk and galacto-oligosaccharides lead to improved skin health by decreasing phenols production by gut microbiota. Benef Microbes. (2014) 5:121–8. 10.3920/bm2012.0066 23685373

[100] Iizuka R Kawakami K Izawa N Chiba K. Phenols produced by gut bacteria affect the skin in hairless mice. Microbial Ecology Health Dis. (2009) 21:50–56. 10.3402/mehd.v21i1.7570

[101] Sugimoto S Ishii Y Izawa N Masuoka N Kano M Sone T . Photoprotective effects of bifidobacterium breve supplementation against skin damage induced by ultraviolet irradiation in hairless mice. Photodermatol Photoimmunol Photomed. (2012) 28:312–9. 10.1111/phpp.12006 23126293

[102] Kawada C Yoshida T Yoshida H Matsuoka R Sakamoto W Odanaka W . Ingested hyaluronan moisturizes dry skin. Nutr J. (2014) 13:70. 10.1186/1475-2891-13-70 25014997 PMC4110621

[103] Yumi N Yuko F Takatsugu T Reiko I. Composition for Promoting Expression of Aquaporin 3. European patent EP3689156A4 (2020).

[104] Komatsu T Sasaki S Manabe Y Hirata T Sugawara T. Preventive effect of dietary astaxanthin on uva-induced skin photoaging in hairless mice. PLoS ONE. (2017) 12:e0171178. 10.1371/journal.pone.0171178 28170435 PMC5295690

[105] Knippschild DS Baulig C Hirsch J Krummenauer F. The consort statement for standardized reporting of randomized clinical trials - evidence as a consequence of transparency. Zeitschrift fur Zahnarztliche Implantologie (2015) 31:64–74.

