# Supplementary material for: Effectiveness of Dietary Supplement for Skin Moisturizing in Healthy Adults: A Systematic Review and Meta-Analysis of Randomized Controlled Trials
Source: Front Nutr. 2022 Jun 2;9:895192. doi: 10.3389/fnut.2022.895192 (PMC9201759; doi:10.3389/fnut.2022.895192)
Supplement: Supplementary file 1 [file Table_1.docx]

**Supplementary Material**

**Supplemental Table S1**. Search strategy used for this review.

| Database | Search strategy | Number of results |
| --- | --- | --- |
| Pubmed | ("Dietary Supplements"[MeSH Terms] OR "Probiotics"[MeSH Terms] OR "plants"[MeSH Terms] OR "food"[MeSH Terms] OR "diet"[MeSH Terms] OR "botanical extract"[All Fields] OR "plant extract"[All Fields] OR "essential oil"[All Fields] OR "phytotherapy"[MeSH Terms] OR "Plant Oils"[MeSH Terms] OR "plant leaves"[MeSH Terms] OR "plant preparations"[MeSH Terms] OR "proteins"[MeSH Terms] OR "ferment*"[All Fields] OR "Lactobacillus"[Title/Abstract] OR "supplement"[Title/Abstract] OR "food"[Title/Abstract] OR "diet*"[Title/Abstract] OR "oral"[Title/Abstract] OR "intake"[Title/Abstract] OR "ingestion"[Title/Abstract]) AND ("skin aging"[MeSH Terms] OR "skin hydration"[All Fields] OR "skin condition"[All Fields] OR "skin moisturizing"[All Fields] OR "skin barrier"[All Fields] OR "Epidermis"[MeSH Terms] OR "Trans Epidermal Water Loss"[All Fields] OR "skin care"[MeSH Terms] OR "dry skin"[All Fields] OR "Wrinkle"[All Fields] OR "skin roughness"[All Fields]) AND (("randomized controlled trial"[Publication Type] OR "controlled clinical trial"[Publication Type] OR "randomized"[Title/Abstract] OR "placebo"[Title/Abstract] OR "clinical trials as topic"[MeSH Terms:noexp] OR "randomly"[Title/Abstract] OR "clinical study"[Title/Abstract] OR "trial"[Title/Abstract] OR "volunteers"[Title/Abstract]) NOT ("animals"[MeSH Terms] NOT ("humans"[MeSH Terms] AND "animals"[MeSH Terms]))) | 1729 |
| Embase | ('dietary supplement'/exp OR 'food'/exp OR 'diet'/exp OR 'probiotic agent'/exp OR 'protein'/exp OR 'plant'/exp OR 'plant extract'/exp OR 'essential oil'/exp OR 'phytotherapy'/exp OR 'vegetable oil'/exp OR 'plant leaf'/exp OR 'plant preparations'/exp OR 'botanical extract' OR 'ferment*' OR 'lactobacillus':ti,ab OR 'supplement':ti,ab OR 'food':ti,ab OR 'diet*':ti,ab OR 'oral':ti,ab OR 'intake':ti,ab OR 'ingestion':ti,ab) AND ('cutaneous parameters'/exp OR 'skin hydration'/exp OR 'skin aging'/exp OR 'skin conditon' OR 'skin moisturizing' OR 'skin barrier'/exp OR 'epidermis'/exp OR 'skin water loss'/exp OR 'trans epidermal water loss' OR 'skin care'/exp OR 'dry skin'/exp OR 'skin roughness'/exp OR 'wrinkle'/exp) AND ('randomized controlled trial':de OR 'controlled clinical trial':de OR 'randomized':ti,ab OR 'placebo':ti,ab OR 'clinical trials' OR 'randomly':ti,ab OR 'clinical study':ti,ab OR 'volunteers':ti,ab) | 5434 |
| Cochrane | ((MeSH descriptor: [Skin Aging] explode all trees) OR (MeSH descriptor: [Epidermis] explode all trees) OR (MeSH descriptor: [Skin Care] explode all trees) OR "dry skin" OR "skin barrier" OR "Skin hydration" OR "Skin Wrinkling" OR "skin roughness" OR "skin condition" OR "skin moisturizing" OR "Trans Epidermal Water Loss" OR "skin water loss" OR Wrinkle) AND ((MeSH descriptor: [Dietary Supplements] explode all trees) OR (MeSH descriptor: [Food] explode all trees) OR (MeSH descriptor: [Diet] explode all trees) OR (MeSH descriptor: [Probiotics] explode all trees) OR (MeSH descriptor: [Plants] explode all trees) OR (MeSH descriptor: [Proteins] explode all trees) OR "botanical extract" OR ferment* OR (MeSH descriptor: [Plant Extracts] explode all trees) OR (MeSH descriptor: [Oils, Volatile] explode all trees) OR (MeSH descriptor: [Phytotherapy] explode all trees) OR (MeSH descriptor: [Plant Oils] explode all trees) OR (MeSH descriptor: [Plant Leaves] explode all trees) OR MeSH descriptor: [Plant Preparations] explode all trees OR (Lactobacillus):ti,ab OR (supplement):ti,ab OR (food):ti,ab OR (diet*):ti,ab OR (oral):ti,ab OR (intake):ti,ab OR (ingestion):ti,ab) | 1400 |

**Supplement Table S2.** Full details of all studies included in systematic review.

| **Study Location** | **Study Population** | | | **Intervention** | **Control** | **Formulation** | **Study Duration** | **Study Periods** | **Test**  **Conditions ( °C R.T.) (% R.H.)** | **Outcome** | | | **Adverse Effects** |
| --- | --- | --- | --- | --- | --- | --- | --- | --- | --- | --- | --- | --- | --- |
|  | **Sample size, sex** | **Age, years** | **Health Condition** | **Contents, daily dose** | **Contents, daily dose** |  |  |  |  | **Meaurement Instrument** | **Parameter (Measuring Sites)** | **Unit** |  |
| Collagen | | | | | | | | | | | | | |
| Asserin 2015 (1);  Japan | n=33;  100% F | 40-59 | Healthy; low skin water content; | 10 g collagen peptides of fish or porcine origin (MW: 2000-5000Da); | 10 g dextrin | beverage | 8w | NR | 22±1;50±10 | Corneometer®; | cheek | au | AE = 0 |
| CHOI 2014 (2); Korea | n=32; 75% F | 30-48 | Healthy | 3 g collagen peptides (MW: 1500Da) or 3g Collagen peptides + 500 mg vitamin C; | Blank control or 500 mg vitamin C; | NR | 12w | NR | NR | Corneometer®; Tewameter®; | cheek | au g/h/m^2^; | NR |
| Inoue 2016 (3); China | n=85; 100% F | 35-55 | no medical issues by blood test; dry and rough skin | 5 g collagen hydrolysate of fish (lower or high content of bioactive collagen peptides) | unclear placebo | beverage | 8w | February to April | 20±2;  50±5 | Corneometer CM820; | cheek; Canthus | au | AE = 0 |
| Kim 2018a (4);  Korea | n=64; 100% F | 40–60 | Healthy subjects with wrinkle(s) in the crow’s-feet area, with global photodamage score between 2 and 6; | 1000 mg Low-molecular-weight Collagen peptide of fish, vitamin C, fruit concentrate mix, flavor mix, excipients, sweetener. | the same formulation except collagen hydrolysate | beverage | 12w | February to June | 22-24; 40-60 | Corneometer CM 825; | cheek | au | AE = 0 |
| Koizumi 2017 (5); Korea. | n=80; 100% F | 30–60 | Healthy women with periorbital wrinkles corresponding to a grade of 2–6; | 3000 mg of Collagen peptides of fish (MW: 3000 Da) | the same ingredient excluded Collagen peptides | beverage | 12w | February to May | 20-24;  40-60 | Corneometer CM 825; | cheek | % | AE = 0 |
| Koizumi 2019 (6)； China | n=85; 100% F | 35-55 | Healthy, awareness of skin roughness and skin dryness; | 5 g collagen hydrolysate (MW: 5000 Da) derived from fish scales or fish skin | Maltodextrin | beverage | 8w | February to April | 20±2;  50±5 | Corneometer CM 820; | cheek; Canthus | au | AE = 0 |
| Maia Campos 2019 (7);  Brazil | n=40; 100% F | 40-50 | Healthy | 9 g hydrolyzed collagen, vitamins A (600 μg), C (45 mg), E (10 mg), and zinc (7,0 mg). | 10 g of maltodextrin | NR | 90d | NR | 21.5±1; 50±5 | Corneometer CM 825; | face | au | NR |
| Miyanaga 2021 (8); Japan | n=99; 100% F | 35-50 | Healthy women with dry skin and decreased skin elasticity; | 1 or 5 g of Collagen peptides (MW: 5000 Da) | A collagen-free beverage (placebo) | beverage | 12w | November to March | 21±2;  45±5 | Corneometer®; Tewameter®; | cheek; forearm | au;  g/h/m^2^; | NR |
| Sugihara 2015 (9);  China | n=56; 100% F | 30-55 | Healthy women who were conscious of their dry and rough skin | 2.5 g of collagen hydrolysate of fish (MW: 1000 Da) | 5 g of maltodextrin | NR | 8w | February to April | 20±2;  50±5 | Corneometer CM 820; | canthus | au | NR |
| Tak 2021 (10);  Korea | n=84; 100% F | 40-60 | Healthy women with a transepidermal water loss (TEWL) score ≥4 | 1,000 mg of collagen tripeptides of fish (MW: 500 Da) | Placebo consisted of 170mg (68.2%) of maltodextrin and 80mg (31.8%) of dextrin | capsule | 12w | NR | 23±1;  45±5 | Corneometer CM 825; Tewameter TM300 | forehead; forearm | au;  g/h/m^2^; | AE = 0 |
| Combination collagen preparations | | | | | | | | | | | | | |
| Bolke 2019 (11);  Germany | n=72; 100% F | 35-73 | Healthy, no major or chronic skin diseases, no major interNRl or chronic diseases | 2.5 g collagen peptides of bovine skin, 666 mg acerola fruit extract, 80 mg vitamin C, 3 mg zinc, 2.3 mg vitamin E, and 50 g biotin; | potassium sorbate, sodium benzoate, carboxymethylcellulose, citric acid, NRtural aroma, and water. | beverage | 12w | NR | 20;  40-60 | Corneometer CM 825; | forearm | au | AE = 0 |
| Ito 2018a (12);  Japan | n=22; 82% F | 31-48 | Healthy; low levels of blood IGF-1; relatively low skin moisture and skin elasticity | 10 g of fish-derived collagen peptides, 400 mg of ornithine, and other ingredients including vitamin C, acidifier, and sweetener | the same amount of vitamin C, acidifier, and sweetener, but did not contain CPO | beverage | 8w | October to December | 21±1;  50±5 | Corneometer; VAPO SCAN; | cheek | au  g/h/m^2^; | AE = 0 |
| Lin 2021 (13);  China | n=50; 100% F | 35-50 | Healthy; yellow-brown skin tone, visible black spots; dry skin; visible wrinkles. | 50 g collagen drink (81% water, 11% fish hydrolyzed collagen, 3% apple juice, 2% Djulis extract) | 95% water, 3% apple juice | beverage | 8w | NR | NR | Corneometer CM825 | cheek | au | AE = 0 |
| Primavera 2005 (14);  Italy | n=32; 100% F | 35-60 | Healthy | sea fish cartilage, vegetable ceramides, amino acids, antioxidants and essential fatty acids | soybean oil | capsule | 40d | NR | 20±2;  45±5 | Corneometer CM 825; | Face | au | NR |
| Schwartz 2019 (15);  USA | n=128; 100% F | 36-59 | Healthy; Fitzpatrick Skin Types I-IV; showed signs of skin aging. | 300 mg of hydrolyzed collagen type-II; 100 mg of glycosaminoglycan, chondroitin sulfate; 50 mg of hyaluronic acid. | 500 mg of cellulose | capsule | 12w | NR | NR | MoistureMeterSC;  VapoMeter; | cheek | NR;  g/h/m^2^; | AE = 0 |
| Žmitek 2020 (16);  Slovenia | n=34; 100% F | 40-65 | Healthy; Fitzpatrick skin phototypes II and III; showed signs of skin aging. | Hydrolyzed fish collagen: 4000 mg, water‐soluble CoQ10: 50 mg, vitamin C: 80 mg, vitamin A: 920 μg, biotin: 150 μg | 10 mL of flavoured and coloured placebo syrup without any active ingredients | beverage | 12w | October to February | 20-25; 40-60 | DermaLab Series, SkinLab Combo | cheek | μs;  g/h/m^2^; | AE = 0 |
| Ceramide | | | | | | | | | | | | | |
| Bizot 2017 (17);  Italy | n=60; 100% F | 30–60 | Healthy; dry skin ; photoaging or mild-to moderate chrono-aging | wheat polar lipids complex-oil form (WPLC-O, 70 mg/d) or wheat polar lipids complex-powder form (WPLC-P, 30 mg/d) | the same ingredient excluded both WPLC-P and WPLC-O | capsule | 60d | NR | 21±1;  50±10 | Corneometer CM 825; Tewameter TM300 | cheek | au;  g/h/m^2^; | NR |
| Boisnic 2019 (18);  France | n=64; 100% F | 45-60 | Healthy women presenting with dry skin on their legs and showing wrinkles of the crow's‐feet area associated with a Lemperle score of between 3 and 5. | Polar lipids from wheat extract oil (WEO) containing 9.5 mg of SLs (ceramides and glycoceramides) | the same ingredient excluded WEO | capsule | 20w | NR | NR | Corneometer CM 825; | face;  leg; | au | AE = 0 |
| Guillou 2011 (19);  France | n=51; 100% F | 20–63 | Healthy women with dry to very dry skin | wheat extract oil (WEO, 350 mg) | starch instead of WEO | capsule | 12w | April to July | 22±2;  50±10 | corneometry (Dermalab , Cortex, Germany) | arm;  leg; | au | AE it=3, pl=4;  TAE NR;  TWD=0; |
| Hori 2010 (20);  Japan | n=35; 100% F | 35-50 | dry skin | 10 mg or 30 mg beet ceramide | the same ingredient excluded beet ceramide | capsule | 8w | January to March | 24.0-24.5;  43.0-49.5 | Corneometer CM 825; Tewameter TM300 | cheek;  arm | au;  g/h/m^2^; | AE = 0 |
| Nishide 2020 (21);  Japan | n=41; 100% F | 32-68 | Healthy | 2mg of phytosphingosine from soy sauce lees | the same ingredient excluded phytosphingosine extract | tablet | 12w | November to February | 22; 25 | Tewameter TM300 | cheek | g/h/m^2^ | AE = 0 |
| Oda 2010 (22);  Japan | n=65; 55% F | 35-56 | Healthy men and women who had mild dry skin and were free of skin diseases such as atopic dermatitis and psoriasis and other diseases with severe xeroderma | 55.6 mg dry Acetobacter malorum NCI 1683 (S24) which contained 400 μg ceramide and 94.4 mg dextrin (low-dose group); or 111.1 mg dry Acetobacter malorum NCI 1683 (S24), which contained 800 μg ceramide and 48.9 mg dextrin (high-dose group). | 120 mg dextrin | capsule | 8w | February to April | 21±1;  45±5 | Corneometer CM 825;  Vapometer; | cheek; forearm;  neck; | au;  g/h/m^2^; | AE = 0 |
| Takara 2021 (23);  Japan | n=44; 100% F | 40-58 | Healthy Japanese female adults (20 years or older) with concerns about skin dehydration | Oryza Ceramide® (40 mg/day) containing 1.2 mg of GlcCer | 200 mg of cyclodextrin | capsule | 12w | August to December | NR | Corneometer CM 825; Tewameter TM300 | cheek | au;  g/h/m^2^; | AE = 0 |
| Tsuchiya 2020a (24); Japan | n=125; 93% F | 20-60 | Healthy individuals with no chronic physical diseases, including skin diseases, | 200 mg of acetic acid bacteria containing 0.8 mg of dihydroceramide | 320 mg dextrin | capsule | 12w | NR | 21±1;  50±5 | Corneometer CM 825; Tewameter TM300 | Cheek  upper arm;  neck; back; | au;  g/h/m^2^; | AE it=23, pl=38;  TAE = 0;  TWD=0; |
| Uchiyama 2008 (25); Japan | n=120; 51% F | 21-59 | Healthy individuals with high TEWL in cheek | Konjac extract containing 1.8 mg glucosylceramide | the same ingredient excluded Konjac extract | beverage | 12w | November to March | 21±2;  45 | Vapometer; | cheek; forearm; back; | g/h/m2; | NR |
| Combination ceramide preparations | | | | | | | | | | | | | |
| Schwartz 2016 (26); USA | n=21; 100% F | 35-65 | Health, baseline Crows’ feet lines and wrinkles grade of ≥2 centimeters (cm) on aten cm Visual Analogue Scale (VAS) at baseline. | Zeaxanthin, sea buckthorn fruit oil, wheat ceramides, alpha lipoic acid, green tea, red clover leaf, gotu kola seed, maritimepine bark, vitamins C, E, D | Safflower oil | NR | 12w | NR | NR | MoistureMeter SC | NR | NR | AE = 0 |
| Primavera 2005 (14);  Italy | n=32; 100% F | 35-60 | Healthy | sea fish cartilage, vegetable ceramides, amino acids, antioxidants and essential fatty acids | soybean oil | capsule | 40d | NR | 20±2;  45±5 | Corneometer CM 825; | Face | au | NR |
| lactic acid bacteria | | | | | | | | | | | | | |
| Chan 2021a (27);  China | n=30; 57% F | 35-55 | Healthy volunteer with yellow-brown skin tone, visible black spots and dry skin | fermented vegetable-fruit juice (Saccharomyces cerevisiae and Streptococcus thermophilus), apple juice, fructose, high methoxyl pectin, citric acid, apple flavor and water. | the same ingredient excluded fermented vegetable-fruit juice | beverage | 8w | NR | NR | Corneometer CM 825; | cheek | au | AE = 0 |
| Chan 2021b (28);  China | n=40; NR | 35-55 | Healthy volunteer with yellow-brown skin tone, visible black spots and dry skin | fermented pomegranate  extract (Saccharomyces cerevisiae and Lactobacillus plantarumto), sucralose, and water; | flavor, citric acid, sucralose, and water | beverage | 8w | NR | NR | Corneometer CM 825; | cheek | au | NR |
| Gueniche 2014 (29);  France | n=64; 100% F | 18-40 | skin-type I/IV, no use of systemic medications | L. paracasei NCC 2461 (ST11) (1×10^10^ cfu,) | maltodextrin | powder | 60d | October to early May | NR | evaporimeter | forearm | AUC | AE NR;  TAE = 0;  TWD=0; |
| Kim 2015 (30);  Korea | n=41; 100% F | 25-60 | Healthy volunteers with dry and dark skin | candy contained 2.1% L. plantarum K8 lysates, | candy contained no lysates | candy | 8w | NR | NR | Corneometer CM825;  Vapometer; | face;  forearm; | au;  g/h/m^2^; | NR |
| Kimoto-Nira 2014 (31); Japan | n=23; 100% F | 19-21 | Healthy | Lactococcus lactis strain H61-fermented milk (1.5x10^10^cfu) | conventional yogurt | beverage | 4w | February to March | 22±25; 33±3 (february); 53±3 (March) | Corneometer CM 825; | cheek; forearm | au | AE = 0 |
| Kimoto-Nira 2015 (32); Japan | n=23; 100% F | 36-62 | Healthy | Lactococcus lactis strain H61-fermented milk (1x10^10^cfu) | conventional yogurt | beverage | 4w | NR | 20-24;  27-39 | corneometer | cheek; forearm | au | AE = 0 |
| Lee 2015 (33);  Korea | n=110; 100% F | 41-59 | Healthy women who have dry skin and wrinkles | 2 g daily of a powder containing Lactobacillus plantarum HY7714 (1x10^10^cfu) | the same product without HY7714. | powder | 12w | NR | 22±2;  50±5 | Corneometer CM 825;  vapometer SWL4001 | cheek; forearm;  hand | au;  g/h/m^2^; | AE = 0 |
| Nagino 2018 (34);  Japan | n=60; 100% F | 18-55 | Healthy premenopausal Japanese women; no medication use, no diseases | 100 ml of fermented soymilk (FSM) with Lactobacillus casei Shirota (40x10^10^cfu) | 100 ml of unfermented placebo soymilk | beverage | 16 w | September to January | NR | self-report questionnaires | face | - | AE NR;  TAE = 0; |
| OGAWA 2016 (35);  Japan | n=118; 72% F | 21-59 | Healthy, relatively higher level of TEWL at the forearm | 0.025 g for the 25 mg/day heat‑killed L. brevis SBC8803 group (group L), and 0.050 g for the 50 mg/day heat‑killed L. brevis SBC8803 group (group H). | the same product without  heat‑killed L. brevis SBC8803 | capsule | 12w | February to June | 20±1;  50±5 | Corneometer CM 825; Tewameter TM300 | forearm; neck;  the lower eye region | au;  g/h/m^2^; | AE NR;  TAE = 0; |
| SAITO 2017 (36);  Japan | n=64; 100% F | 20-64 | Healthy women who had relatively high rates of TEWL in pretrial testing and relatively low stool frequency | Lactobacillus casei subsp. casei 327 (50 mg), glucose, starch, calcium stearate, fine granular silica, hydroxypropylcellulose | the same ingredient excluded Lactobacillus casei subsp. casei 327 | tablet | 8w | NR | 21±1;  50±5 | Corneometer CM 825; Tewameter TM300 | cheek; upper arm | au;  g/h/m^2^; | AE it=14, pl=12;  TAE = 0;  TWD NR; |
| Combination lactic acid bacteria preparation | | | | | | | | | | | | | |
| Choi 2018 (37);  Korea | n=120; 57% F | 35-60 | Healthy Korean subjects have moderate to severe crow’s feet wrinkles | 100 mg or 200 mg fermented honeybush extract (Streptococcus thermophilus) | dextran | capsule | 12w | NR | NR | Corneometer®; Tewameter®; | cheek | au;  g/h/m^2^; | AE = 0 |
| KANO 2013 (38);  Japan | n=40; 100% F | 23-75 | Healthy | fermented milk containing Bifidobacterium breve strain Yakult (6x10^10^cfu) and galacto-oligosaccharides (GOS) | non-fermented placebo milk containing neither probiotics nor GOS | beverage | 8w | October to December | 23±1;  45±5 | Skicon® 200 | cheek | μs | NR |
| Mori 2016 (39);  Japan | n=101; 100% F | 18-23 | Healthy | a fermented milk containing GOS, polydextrose, B. breve strain Yakult (YIT 12272), Lactococcus lactis YIT 2027 (>1x10^10^ cfu), and Streptococcus thermophilus YIT 2021 (>1x10^10^ cfu) | blank control | beverage | 8w | October to December | 21.9±0.7;  48.7±1.5 | Corneometer® CM825 | cheek; | au | NR |
| Puch 2008 (40);  France | n=72; 100% F | 20-45 | Healthy women with skin phototype I–III, showing no signs of skin diseases, | two daily doses of 150 mg GLA in BO, 47 mg catechins and 2 mg vitamin E, mixed in a dairy matrix containing probiotics (Lactobacillus casei, Lactobacillus bulgaricus and Streptococcus thermophilus) | acidified milk without GLA in BO, catechins, vitamin E and probiotics | beverage | 24w | NR | 20-22;  30-40 | Evaporimeter | forearm | g/h/m^2^; | NR |
| Hyaluronan | | | | | | | | | | | | | |
| Hsu 2021 (41);  China | n=40; 73% F | 35-64 | Healthy, not currently under-going any medical treatment | 120 mg/capsule/day of HA (Hyabest®(S)LF-P; Kewpie Corporation, Tokyo, Japan) | dextrin | capsule | 12w | May to August | 20±2;  50±5 | Corneometer CM 825; Tewameter TM300 | face; Arm;  Waist | au;  g/h/m^2^; | AE NR;  TAE = 0; |
| Kawada 2015 (42): Japan | n=61; 100% F | 35-60 | Health women with dry skin | Oral HA (120 mg/day), of MWs 800K and 300K | 540 mg of Cellulose | capsule | 8w | NR | 21±2;  50±10 | Corneometer CM 825; | cheek; | au | AE = 0 |
| Michelotti 2021 (43);  Italy | n=60; 100% F | 35-70 | Healthy; showing mild to moderate sign of skin aging | 200-mg daily dose of FSHA (full-spectrum hyaluronan) | 200 mg of maltodextrin | NR | 4w | September to November | 22±2;  40-60 | Corneometer CM 825; Tewameter TM300 | face | au;  g/h/m^2^; | AE = 0 |
| SATO2002 (44);  Japan | n=35;63% F | 18-45 | Health subjects with dry skin | Oral HA (120 mg/day) | 200 mg of Cellulose | capsule | 4w | NR | 20.5-23.5; 45-60 | Corneometer CM 825; | upper arm;  the lower eye region;  Back of neck | au | AE = 0 |
| Polyphenols | | | | | | | | | | | | | |
| Buonocore 2012 (45); Italy | n=50; 70% F | 35-65 | Health and absence of skin diseases; showed clinical signs of skin aging (wrinkles, dull complexion, brown spots). | 8 mg transresveratrol, 14.63 mg procyanidins, 0.67 mg anthocyanosides, 0.4 mg flavonoids, 1.3 mg other stilbenes, 3.75 mg procyanidins, 8.75 mg punicalagin-ellagic acid, 50 μg selenium, 26 mg silicon dioxide, and 45 mg maltodextrin. | 266 mg dehydrated dibasic calcium phosphate, 18 mg pigment red iron oxide (E 172), 4 mg magnesium stearate, 4 mg amorphous precipitated silica, and 77 mg activated charcoal. | capsule | 60d | NR | NR | Corneometer CM 825; | cheek; | au | NR |
| Hughes-Formella 2007 (46);  Germany | n=42; 76% F | 19-49 | Healthy; skin type I–III according to Fitzpatrick | Anthogenol products derived from V. vinifera (100 mg/day) | blank control | NR | 6w | NR | 20±2;  50±10 | Corneometer CM 825; | forearm | au | NR |
| Shoji 2020 (47);  Janpan | n=65; 100% F | 20-39 | Healthy women participants with skin photo-type-II and -III | Apple Polyphenol (300 or 600 mg/day) | 600 mg starch decomposition product/day. | tablet | 12w | January to April | 20±1;  45±5 | Skicon-200 EX; Vapo Scan AS-VT100RS | corner of the eye | au; g/h/m^2^; | AE NR;  TAE = 0; |
| Tsuchiya 2020b (48); Japan | n=100; 100% F | 30-59 | Healthy women with measurable sunspots on cheeks | the test beverage (200 mL) contained 200 mg of the red wine OPCs (grapes oligomeric procyanidins) | the control beverage (200 mL) did not contain red wine OPCs | beverage | 12w | November to February | 21±1;  50±5 | Corneometer CM825 | cheek; | au | AE it=13, pl=10;  TAE = 0;  TWD = 0; |
| Heinrich 2006 (49); Germany | n=24; 100% F | 18-65 | Healthy, normal skin of type II, as described by Fitzpatrick and Pathak | high flavanol (326 mg/d) cocoa powder dissolved in 100 mL water | low flavanol (27 mg/d) cocoa powder dissolved in 100 mL water | beverage | 12w | NR | NR | Corneometer CM 825; Tewameter TM300 | face | au;  g/h/m^2^; | NR |
| Mogollon 2014 (50); Canada | n=74; 100% F | 20-65 | Healthy, normal skin types I or II, as described by Fitzpatrick | High-flavanol chocolate containing 600 mg of flavanols daily | Low-flavanol chocolate containing < 90 mg of flavanols daily | chocolate | 12w | NR | 22±2;  40-60 | Corneometer CM825 | Temple;  Arm | au | AE = 0 |
| Fukagawa 2017 (51); Japan | n=54; 100% F | 25-40 | Healthy subjects with a relatively high dryness score and a low skin hydration of the lower cheek | Coffee polyphenols (CPPs)(270 mg/100 mL/day) | the same ingredient  excluded CPPs | beverage | 8w | November to February | 20±2;  40±5 | Corneometer CM 825; Tewameter TM300 | cheek;  hand | %;% | AE = 0 |
| Tseng 2021 (52);  China | n=40; 100% F | 35-55 | Healthy, yellow-brown skin tone, visible black spots, dry skin, visible wrinkles. | 50 ml of coffee pulp drink (CPD) | the same ingredient  excluded CPD | beverage | 8w | NR | NR | Corneometer CM825 | cheek | au | NR |
| Heinrich 2011 (53); Germany | n=60; 100% F | 40-65 | Healthy, normal skin of type II according to Fitzpatrick | 1 L of green tea beverage (1402 mg total tea catechins) | control beverage excluded green tea extract | beverage | 12w | May to  September | NR | Corneometer CM 825; Tewameter TM300 | forearm | au;  g/h/m^2^; | NR |
| Carotenoids: Astaxanthin | | | | | | | | | | | | | |
| Ito 2018b (54);  Japan | n=23; 91% F | 30-56 | Healthy, skin phototype of type II or type III | 4 mg of astaxanthin | The placebo capsule contained a filling agent instead of astaxanthin. | capsule | 10w | October to December | 21±1;  50±5 | Corneometer; VAPOSCAN AS-VT100RS | The back skin | au;  g/h/m^2^; | AE = 0; |
| Phetcharat 2015 (55); Thailand | n=34; NR | 35-65 | Health; free from any known diseases; having well-defined crow’s-feet wrinkles or possibly other well-defined wrinkles on the face | 4 mg astaxanthin | 3 g of FDA approved dietary supplement – rose hip powder with seeds and shells | capsule | 8w | NR | 25; NR | Corneometer CM825 | forehead | au | NR |
| Yamashita 2006 (56); Japan | n=49; 100% F | Mean age of 47 years | Healthy; (9 dry skin,1 oily skin,9 normal skin,and 30 comboskin) | H. pluvialis (containing 2 mg astaxanthin)  + canola oil | canola oil | capsule | 6w | From January | 20; 65 | Dermal Phase Meter 9003 (NOVA meter) | cheek | % | NR |
| Combination astaxanthin preparation | | | | | | | | | | | | | |
| Yamashita 2002 (57); Japan | n=16; 100% F | Mean age of 40 years | Healthy with dry skin | 2 mg of astaxanthin and 40 mg of tocotrienol | canola oil | capsule | 8w | From January | 20; 65 | Electrical conductance-type SKICON-200 (IBS)). | cheek; the right side outer corners of the eye | μs | NR |
| Yoon 2014 (58);  Korea | n=44; 100% F | 41–60 | Healthy, skin wrinkles grade ≥ 2 | astaxanthin (2 mg) combined with collagen hydrolysate (3 g) | medium chain triglycerides (500 mg per capsule) and tablets with hydrolyzed casein (0.75 g per tablet) | capsule, tablets | 12w | NR | 20–25; 45–55 | Corneometer ; Tewameter | cheek | au;  g/h/m^2^; | AE = 0 |
| Other Carotenoids preparation | | | | | | | | | | | | | |
| Palombo 2007 (59); Italy | n=40; 100% F | 25-50 | Healthy; exhibited signs of premature skin aging | lutein 10 mg and zeaxanthin 0.6 mg | butylene glycol | capsule | 12w | NR | 22;  25 | 3C System methodology | NR | NR | AE = 0 |
| Schwartz 2016 (26); USA | n=21; 100% F | 35-65 | health, baseline Crows’ feet lines and wrinkles grade of ≥2 centimeters (cm) on a ten cm Visual Analogue Scale (VAS) at baseline. | Zeaxanthin, sea buckthorn fruit oil, wheat ceramides, alpha lipoic acid, green tea, red clover leaf, gotu kola seed, maritime pine bark, vitamins C, E, D3 | Safflower oil | NR | 12w | NR | NR | MoistureMeter SC | NR | NR | AE = 0 |
| Aloe | | | | | | | | | | | | | |
| Kaminaka 2020 (60); Japan | n=122; 100% F | 30-55 | Healthy volunteers who might have had minor skin problems including dryness/scales or acne, but did not require medical treatment | 0.25 g of Aloe sterol-containing AVGE (Aloe vera gel extract) powder | the AVGE powder was replaced with starch | capsule | 12w | NR | 20-22;  45-55 | Corneometer CM 825; Tewameter TM300 | forearm | au;  g/h/m^2^; | AE it=129,  pl=111;  TAE = 0;  TWD = 0; |
| Tanaka 2015 (61);  Japan | n=58; 100% F | 20-50 | Healthy; dry skin | 0.5 g aloe vera gel powder (AVGP) containing 40 μg of Aloe sterol | AVGP was replaced by inert dextrin | tablet | 8w | September to December | 20-22;  45-55 | Corneometer CM 825 | upper arm; cheek. | au | AE NR;  TAE = 0; |
| Tanaka 2016 (62);  Japan | n=64; 100% F | 30–59 | Healthy | Aloe sterol yogurt containing 40 μg of Aloe sterol | Aloe vera gel powder was replaced with guar gum | beverage | 12w | August to December | 20-22;  45-55 | Corneometer CM 825; Tewameter TM300 | forearm | au;  g/h/m^2^; | AE NR;  TAE = 0; |
| Turmeric | | | | | | | | | | | | | |
| Vaughn 2018 (63);  USA | n=30; 90% F | 25-60 | Healthy | turmeric tablets contained 500 mg certified organic turmeric root (C. longa). | Banyan Botanicals | tablet | 4w | August to July | NR | VapoMeter; | forehead; cheek | g/h/m^2^; | AE = 0 |
| Asada 2019 (64);  Japan | n=45; 67% F | Mean age of 41 years | Healthy | water extract of Curcuma longa with or without curcumin | maltose and sugar ester | tablet | 8w | January to March | 21±1;  50±5 | Skicon‐200EX; ASVT100RS | cheek | au;  g/h/m^2^; | AE it=28, pl=30;  TAE = 0; |
| Porcine Placenta Extract | | | | | | | | | | | | | |
| Nagae 2020 (65);  Japan | n=20; 100% F | 40–59 | Healthy | 200 mg of porcine placenta extract once daily | 211 mg of dextrin once daily | tablet | 4w | January to February | 23±5;  50±15 | Corneometer CM 825; Tewameter TM300 | upper arm; cheek. | au;  g/h/m^2^; | AE = 0 |
| Kim 2018b (66);  Japan | n=45; 86% F | 30-60 | Healthy; dry skin | Porcine Placenta Extract; MCT oil; fish oil; olive oil and other kinds of oils | MCT oils; gelatin; glycerin | capsule | 8w | February to April | NR | Corneometer CM 825; Tewameter TM300 | upper arm; cheek. | au;  g/h/m^2^; | AE = 0 |

F = female; NR = not reported; AE = adverse events; it = intervention; pl = placebo; TAE = treatment-related adverse effect; TWD = treatment-related withdrawals;


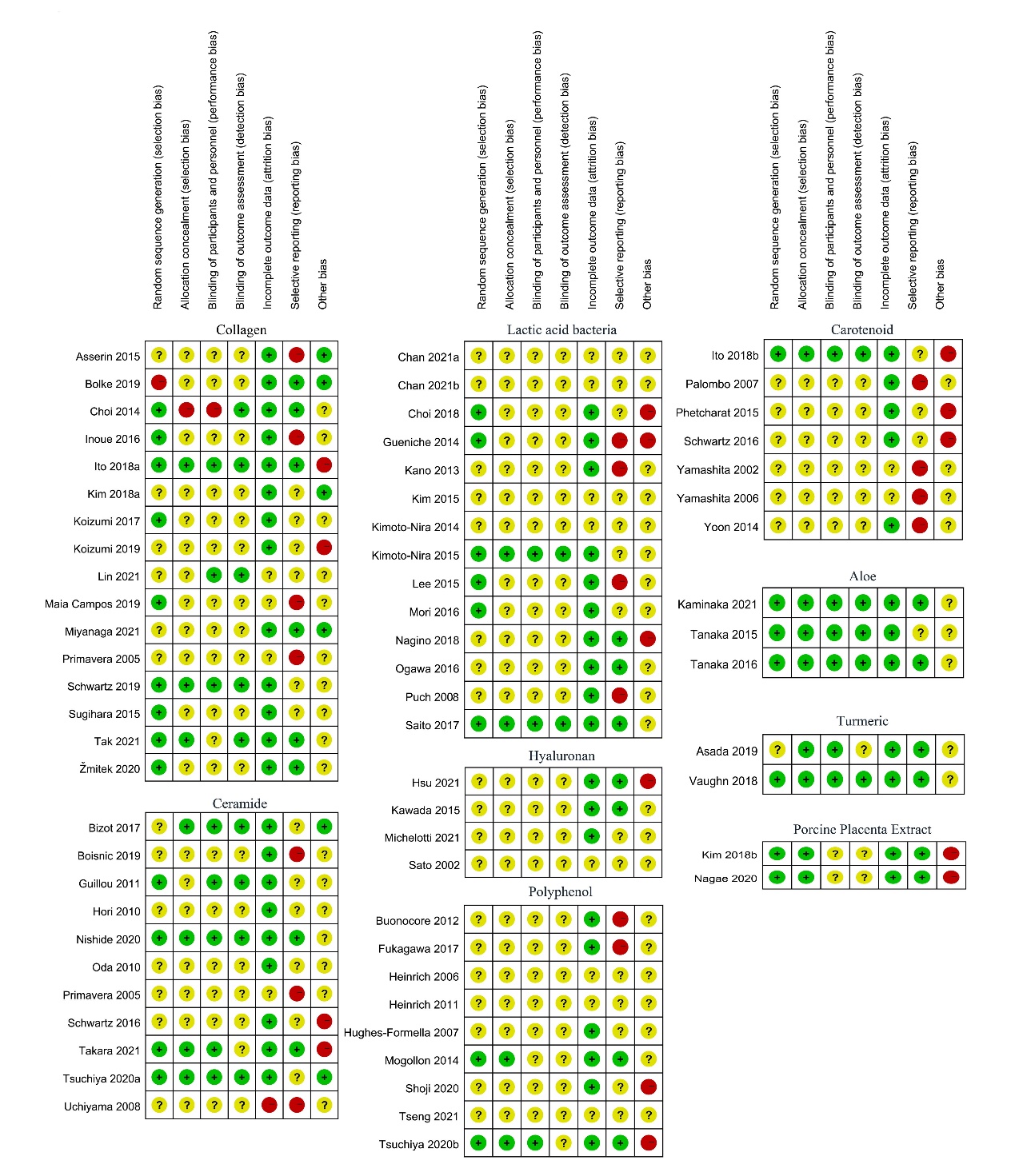


**Supplemental Figure S1** Risk of bias summary: review authors’ judgements about each risk of bias domain for each included study. + = low risk of bias; ? = unclear risk of bias; - = high risk of bias


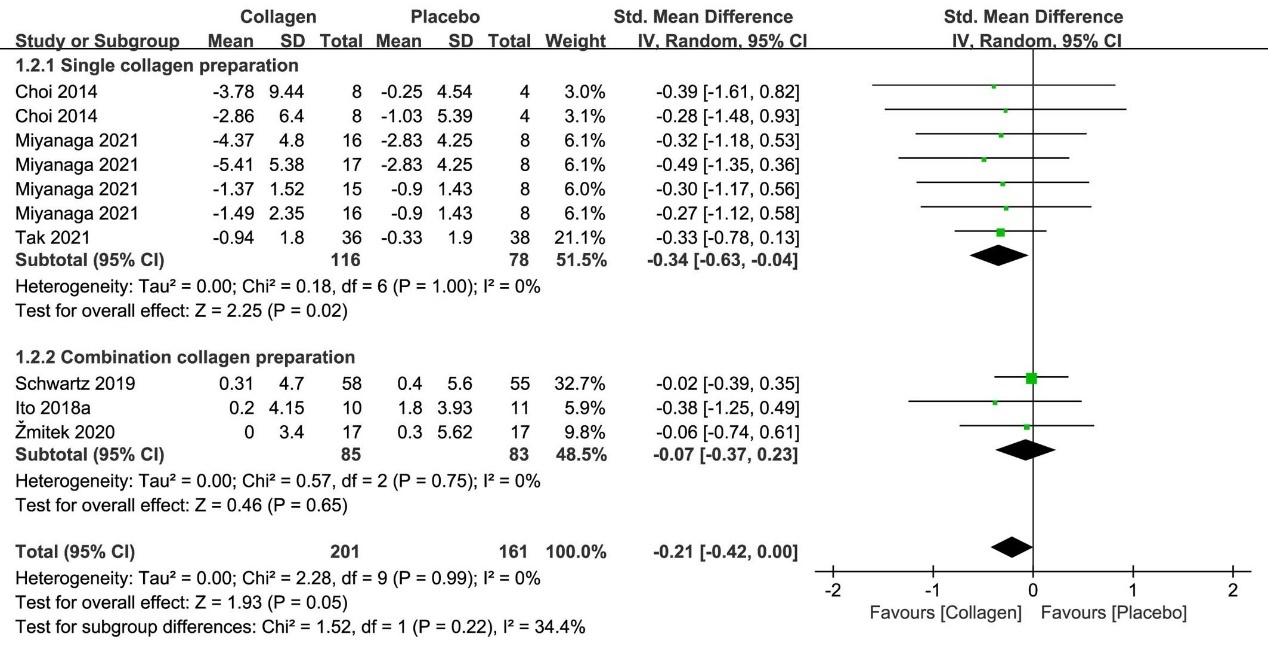


**Supplemental Figure S2** Forest plot of comparison: Collagen vs placebo on TEWL (SMD). The details of each study are reported in Table S2. CI, confidence interval.


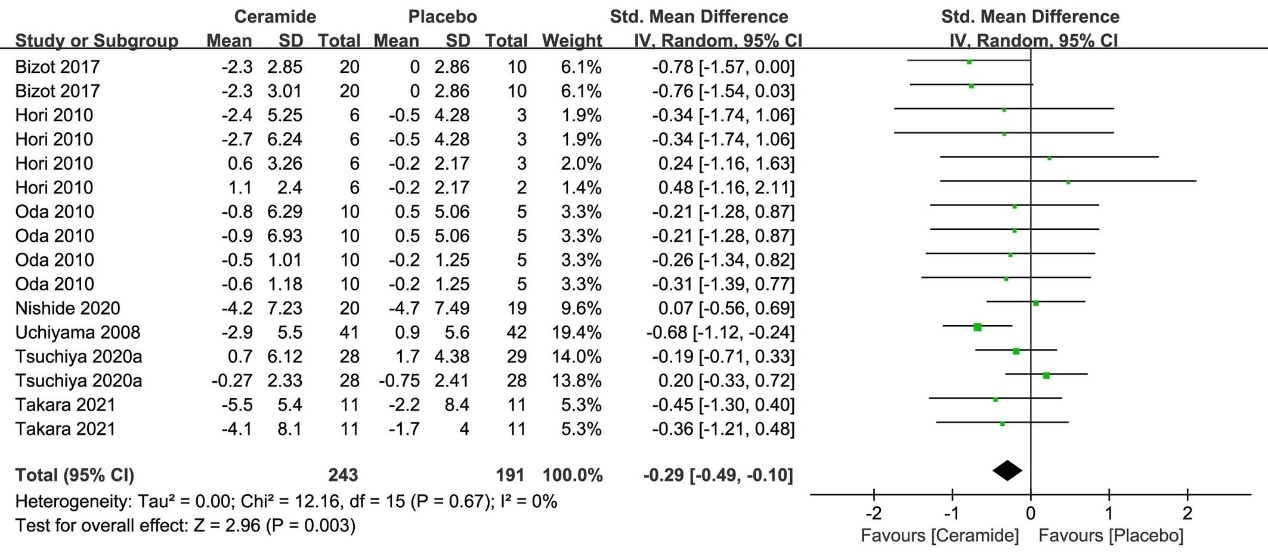


**Supplemental Figure S3** Forest plot of comparison: Ceramide vs placebo on TEWL (SMD). The details of each study are reported in Table S2. CI, confidence interval


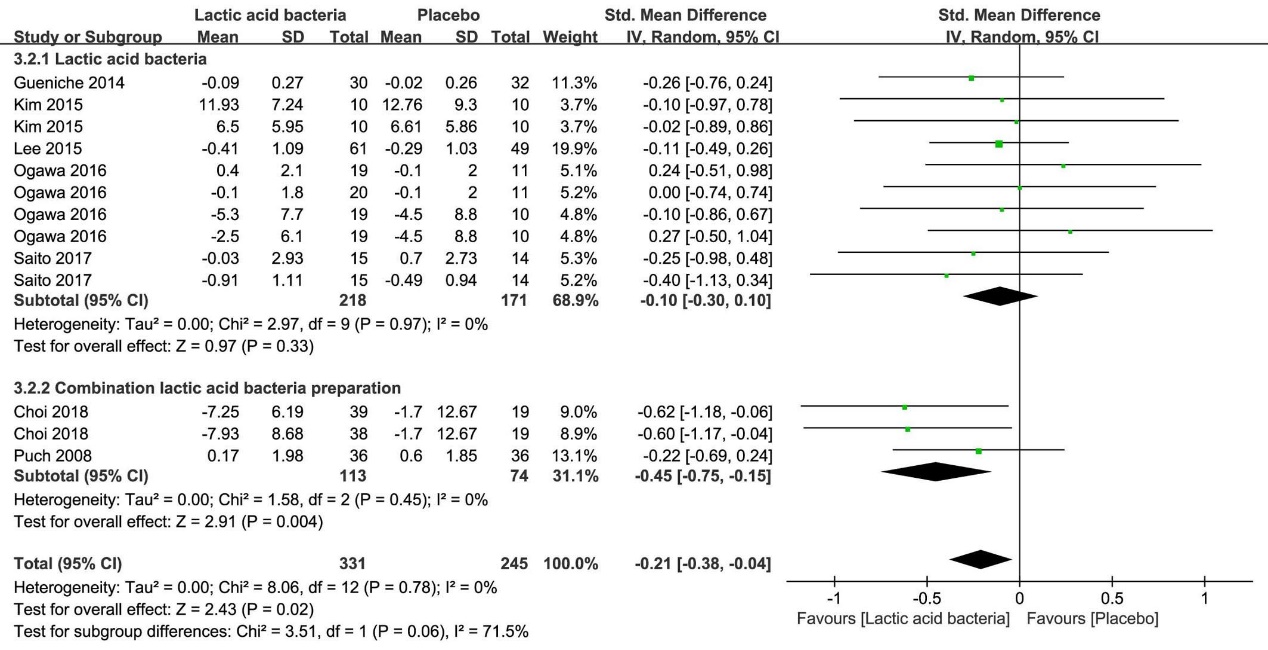


**Supplemental Figure S4** Forest plot of comparison: Lactic acid bacteria vs placebo on TEWL (SMD). The details of each study are reported in Table S2. CI, confidence interval.

**References (Table S2)**

1. Asserin J, Lati E, Shioya T, Prawitt J. The Effect of Oral Collagen Peptide Supplementation on Skin Moisture and the Dermal Collagen Network: Evidence from an Ex Vivo Model and Randomized, Placebo-Controlled Clinical Trials. *J Cosmet Dermatol* (2015) 14(4):291-301. doi: 10.1111/jocd.12174.

2. Choi SY, Ko EJ, Lee YH, Kim BG, Shin HJ, Seo DB, et al. Effects of Collagen Tripeptide Supplement on Skin Properties: A Prospective, Randomized, Controlled Study. *Journal of cosmetic and laser therapy : official publication of the European Society for Laser Dermatology* (2014) 16(3):132-137. doi: 10.3109/14764172.2013.854119.

3. Inoue N, Sugihara F, Wang X. Ingestion of Bioactive Collagen Hydrolysates Enhance Facial Skin Moisture and Elasticity and Reduce Facial Ageing Signs in a Randomised Double-Blind Placebo-Controlled Clinical Study. *J Sci Food Agric* (2016) 96(12):4077-4081. doi: 10.1002/jsfa.7606.

4. Kim DU, Chung HC, Choi J, Sakai Y, Lee BY. Oral Intake of Low-Molecular-Weight Collagen Peptide Improves Hydration, Elasticity, and Wrinkling in Human Skin: A Randomized, Double-Blind, Placebo-Controlled Study. *Nutrients* (2018) 10(7). doi: 10.3390/nu10070826.

5. Koizumi S, Inoue N, Shimizu M, Kwon C-j, Kim H-y, Park K. Effects of Dietary Supplementation with Fish Scales-Derived Collagen Peptides on Skin Parameters and Condition: A Randomized, Placebo-Controlled, Double-Blind Study. *International Journal of Peptide Research and Therapeutics* (2017) 24. doi: 10.1007/s10989-017-9626-0.

6. Koizumi S, Sugihara F, Inoue N, Wang X. The Effects of Collagen Hydrolysates Derived from Tilapia Scales or Skin on Human Facial Skin-a Randomized Double-Blind Placebo-Controlled Clinical Study. *Japanese Pharmacology and Therapeutics* (2019) 47:57-63.

7. Maia Campos P, Melo MO, Siqueira César FC. Topical Application and Oral Supplementation of Peptides in the Improvement of Skin Viscoelasticity and Density. *J Cosmet Dermatol* (2019) 18(6):1693-1699. doi: 10.1111/jocd.12893.

8. Miyanaga M, Uchiyama T, Motoyama A, Ochiai N, Ueda O, Ogo M. Oral Supplementation of Collagen Peptides Improves Skin Hydration by Increasing the Natural Moisturizing Factor Content in the Stratum Corneum: A Randomized, Double-Blind, Placebo-Controlled Clinical Trial. *Skin pharmacology and physiology* (2021) 34(3):115-127. doi: 10.1159/000513988.

9. Sugihara F, Inoue N, Wang X. Clinical Effects of Ingesting Collagen Hydrolysate on Facial Skin Properties: -a Randomized, Placebo-Controlled, Double-Blind Trial. *Japanese Pharmacology and Therapeutics* (2015) 43:67-70.

10. Tak YJ, Shin DK, Kim AH, Kim JI, Lee YL, Ko HC, et al. Effect of Collagen Tripeptide and Adjusting for Climate Change on Skin Hydration in Middle-Aged Women: A Randomized, Double-Blind, Placebo-Controlled Trial. *Frontiers in medicine* (2021) 7:608903. doi: 10.3389/fmed.2020.608903.

11. Bolke L, Schlippe G, Gerß J, Voss W. A Collagen Supplement Improves Skin Hydration, Elasticity, Roughness, and Density: Results of a Randomized, Placebo-Controlled, Blind Study. *Nutrients* (2019) 11(10). doi: 10.3390/nu11102494.

12. Ito N, Seki S, Ueda F. Effects of Composite Supplement Containing Collagen Peptide and Ornithine on Skin Conditions and Plasma Igf-1 Levels-a Randomized, Double-Blind, Placebo-Controlled Trial. *Marine drugs* (2018) 16(12). doi: 10.3390/md16120482.

13. Lin P, Alexander RA, Liang CH, Liu C, Lin YH, Lin YH, et al. Collagen Formula with Djulis for Improvement of Skin Hydration, Brightness, Texture, Crow's Feet, and Collagen Content: A Double-Blind, Randomized, Placebo-Controlled Trial. *J Cosmet Dermatol* (2021) 20(1):188-194. doi: 10.1111/jocd.13500.

14. Primavera G, Berardesca E. Clinical and Instrumental Evaluation of a Food Supplement in Improving Skin Hydration. *International journal of cosmetic science* (2005) 27(4):199-204. doi: 10.1111/j.1467-2494.2005.00237.x.

15. Schwartz SR, Hammon KA, Gafner A, Dahl A, Guttman N, Fong M, et al. Novel Hydrolyzed Chicken Sternal Cartilage Extract Improves Facial Epidermis and Connective Tissue in Healthy Adult Females: A Randomized, Double-Blind, Placebo-Controlled Trial. *Alternative therapies in health and medicine* (2019) 25(5):12-29.

16. Žmitek K, Žmitek J, Rogl Butina M, Pogačnik T. Effects of a Combination of Water-Soluble Coenzymeq10 and Collagen on Skin Parameters and Condition:Results of a Randomised, Placebo-Controlled,Double-Blind Study. *Nutrients* (2020) 12(3). doi: 10.3390/nu12030618.

17. Bizot V, Cestone E, Michelotti A, Nobile V. Improving Skin Hydration and Age-Related Symptoms by Oral Administration of Wheat Glucosylceramides and Digalactosyl Diglycerides: A Human Clinical Study. *Cosmetics* (2017) 4:37. doi: 10.3390/cosmetics4040037.

18. Boisnic S, Keophiphath M, Serandour AL, Branchet MC, Le Breton S, Lamour I, et al. Polar Lipids from Wheat Extract Oil Improve Skin Damages Induced by Aging: Evidence from a Randomized, Placebo-Controlled Clinical Trial in Women and an Ex Vivo Study on Human Skin Explant. *J Cosmet Dermatol* (2019) 18(6):2027-2036. doi: 10.1111/jocd.12967.

19. Guillou S, Ghabri S, Jannot C, Gaillard E, Lamour I, Boisnic S. The Moisturizing Effect of a Wheat Extract Food Supplement on Women's Skin: A Randomized, Double-Blind Placebo-Controlled Trial. *International journal of cosmetic science* (2011) 33(2):138-143. doi: 10.1111/j.1468-2494.2010.00600.x.

20. Hori M, Kishimoto S, Tezuka Y, Nishigori H, Nomoto K, Hamada U, et al. Double-Blind Study on Effects of Glucosyl Ceramide in Beet Extract on Skin Elasticity and Fibronectin Production in Human Dermal Fibroblasts. *Anti-Aging Medicine* (2010) 7:129-142. doi: 10.3793/jaam.7.129.

21. Nishidc A, Shimura, M., Ohnuki, K., Shimizu, K., & Ohnukr, K. Effects of Oral Use of Phytosphingosine on Skin Moisturizing in Healthy Adults-a 12-Week Double-Blind, Randomized, Placebo-Controlled Trial. *Japanese Pharmacology and Therapeutics* (2020) 48(2):237-241.

22. Oda T, Tachimoto H, Kishi M, Kaga T, Ichihashi M. Effect of Oral Intake of Ceramide-Containing Acetic Acid Bcteria on Skin Barrier Function. *Anti-aging Medicine* (2010) 7:50-54. doi: 10.3793/jaam.7.50.

23. Takara T, Yamamoto K, Suzuki N, Yamashita S, Iio S-i, Noguchi H, et al. Oryza Ceramide®, a Rice-Derived Extract Consisting of Glucosylceramides and Β-Sitosterol Glucoside, Improves Facial Skin Dehydration in Japanese Subjects. *Functional Foods in Health and Disease* (2021) 11:385. doi: 10.31989/ffhd.v11i8.809.

24. Tsuchiya Y, Ban M, Kishi M, Ono T. Safety Evaluation of the Excessive Intake of Ceramide-Containing Acetic Acid Bacteria - a Randomized, Double-Blind, Placebo-Controlled Study over a 4-Week Period. *J Oleo Sci* (2020) 69(11):1497-1508. doi: 10.5650/jos.ess20198.

25. Uchiyama T, Nakano Y, Ueda O, Mori H, Nakashima M, Noda A, et al. Oral Intake of Glucosylceramide Improves Relatively Higher Level of Transepidermal Water Loss in Mice and Healthy Human Subjects. *Journal of Health Science - J HEALTH SCI* (2008) 54:559-566. doi: 10.1248/jhs.54.559.

26. Schwartz S, Frank E, Gierhart D, Simpson P, Frumento R. Zeaxanthin-Based Dietary Supplement and Topical Serum Improve Hydration and Reduce Wrinkle Count in Female Subjects. *J Cosmet Dermatol* (2016) 15(4):e13-e20. doi: 10.1111/jocd.12226.

27. Chan LP, Tseng YP, Liu C, Liang CH. Fermented Pomegranate Extracts Protect against Oxidative Stress and Aging of Skin. *J Cosmet Dermatol* (2021). doi: 10.1111/jocd.14379.

28. Chan LP, Tseng YP, Liu C, Liang CH. Anti-Oxidant and Anti-Aging Activities of Fermented Vegetable-Fruit Drink. *Journal of Food and Nutrition Research* (2021) 9:240-250. doi: 10.12691/jfnr-9-5-1.

29. Gueniche A, Philippe D, Bastien P, Reuteler G, Blum S, Castiel-Higounenc I, et al. Randomised Double-Blind Placebo-Controlled Study of the Effect of Lactobacillus Paracasei Ncc 2461 on Skin Reactivity. *Beneficial microbes* (2014) 5(2):137-145. doi: 10.3920/bm2013.0001.

30. Kim H, Kim HR, Jeong BJ, Lee SS, Kim TR, Jeong JH, et al. Effects of Oral Intake of Kimchi-Derived Lactobacillus Plantarum K8 Lysates on Skin Moisturizing. *Journal of microbiology and biotechnology* (2015) 25(1):74-80. doi: 10.4014/jmb.1407.07078.

31. Kimoto-Nira H, Nagakura Y, Kodama C, Shimizu T, Okuta M, Sasaki K, et al. Effects of Ingesting Milk Fermented by Lactococcus Lactis H61 on Skin Health in Young Women: A Randomized Double-Blind Study. *Journal of dairy science* (2014) 97(9):5898-5903. doi: 10.3168/jds.2014-7980.

32. Kimoto-Nira H, Moriya N, Sasaki K, Suzuki C. Effects of Ingesting Milk Fermented by Lactococcus Lactis H61 on Skin Properties and Health Biomarkers in Middle-Aged Women: A Randomized, Double-Blind Study. Journal of Aging Research and Clinical Practice (2015) 4: 109-115. doi: 10.14283/jarcp.2015.57.

33. Lee DE, Huh CS, Ra J, Choi ID, Jeong JW, Kim SH, et al. Clinical Evidence of Effects of Lactobacillus Plantarum Hy7714 on Skin Aging: A Randomized, Double Blind, Placebo-Controlled Study. *Journal of microbiology and biotechnology* (2015) 25(12):2160-2168. doi: 10.4014/jmb.1509.09021.

34. Nagino T, Kaga C, Kano M, Masuoka N, Anbe M, Moriyama K, et al. Effects of Fermented Soymilk with Lactobacillus Casei Shirota on Skin Condition and the Gut Microbiota: A Randomised Clinical Pilot Trial. *Beneficial microbes* (2018) 9(2):209-218. doi: 10.3920/bm2017.0091.

35. Ogawa M, Saiki A, Matsui Y, Tsuchimoto N, Nakakita Y, Takata Y, et al. Effects of Oral Intake of Heat-Killed Lactobacillus Brevis Sbc8803 (Sbl88™) on Dry Skin Conditions: A Randomized, Double-Blind, Placebo-Controlled Study. *Exp Ther Med* (2016) 12(6):3863-3872. doi: 10.3892/etm.2016.3862.

36. Saito Y, Mihara T, Maruyama K, Saito J, Ikeda M, Tomonaga A, et al. Effects of Intake of Lactobacillus Casei Subsp. Casei 327 on Skin Conditions: A Randomized, Double-Blind, Placebo-Controlled, Parallel-Group Study in Women. *Bioscience of microbiota, food and health* (2017) 36(3):111-120. doi: 10.12938/bmfh.16-031.

37. Choi SY, Hong JY, Ko EJ, Kim BJ, Hong SW, Lim MH, et al. Protective Effects of Fermented Honeybush (Cyclopia Intermedia) Extract (Hu-018) against Skin Aging: A Randomized, Double-Blinded, Placebo-Controlled Study. *Journal of cosmetic and laser therapy : official publication of the European Society for Laser Dermatology* (2018) 20(5):313-318. doi: 10.1080/14764172.2017.1418512.

38. Kano M, Masuoka N, Kaga C, Sugimoto S, Iizuka R, Manabe K, et al. Consecutive Intake of Fermented Milk Containing Bifidobacterium Breve Strain Yakult and Galacto-Oligosaccharides Benefits Skin Condition in Healthy Adult Women. *Bioscience of microbiota, food and health* (2013) 32(1):33-39. doi: 10.12938/bmfh.32.33.

39. Mori N, Kano M, Masuoka N, Konno T, Suzuki Y, Miyazaki K, et al. Effect of Probiotic and Prebiotic Fermented Milk on Skin and Intestinal Conditions in Healthy Young Female Students. *Bioscience of microbiota, food and health* (2016) 35(3):105-112. doi: 10.12938/bmfh.2015-022.

40. Puch F, Samson-Villeger S, Guyonnet D, Blachon JL, Rawlings AV, Lassel T. Consumption of Functional Fermented Milk Containing Borage Oil, Green Tea and Vitamin E Enhances Skin Barrier Function. *Exp Dermatol* (2008) 17(8):668-674. doi: 10.1111/j.1600-0625.2007.00688.x.

41. Hsu TF, Su ZR, Hsieh YH, Wang MF, Oe M, Matsuoka R, et al. Oral Hyaluronan Relieves Wrinkles and Improves Dry Skin: A 12-Week Double-Blinded, Placebo-Controlled Study. *Nutrients* (2021) 13(7). doi: 10.3390/nu13072220.

42 Kawada C, Yoshida T, Yoshida H, Sakamoto W, Odanaka W, Sato T, et al. Ingestion of Hyaluronans (Molecular Weights 800 K and 300 K) Improves Dry Skin Conditions: A Randomized, Double Blind, Controlled Study. *Journal of clinical biochemistry and nutrition* (2015) 56(1):66-73. doi: 10.3164/jcbn.14-81.

43. Michelotti A, Cestone E, De Ponti I, Pisati M, Sparta E, Tursi F. Oral Intake of a New Full-Spectrum Hyaluronan Improves Skin Profilometry and Ageing: A Randomized, Double-Blind, Placebo-Controlled Clinical Trial. *European journal of dermatology: EJD* (2021) 31(6):798-805. doi: 10.1684/ejd.2021.4176.

44. Sato T, Sakakoto W, Odanaka W, Yoshida K, Urishibata O. Clinical effects of dietary hyaluronic acid on dry, rough skin. *Aesthetic dermatology* (2002) 12:109-120.

45. Buonocore D, Lazzeretti A, Tocabens P, Nobile V, Cestone E, Santin G, et al. Resveratrol-Procyanidin Blend: Nutraceutical and Antiaging Efficacy Evaluated in a Placebocontrolled, Double-Blind Study. *Clinical, cosmetic and investigational dermatology* (2012) 5:159-165. doi: 10.2147/ccid.S36102.

46. Hughes-Formella B, Wunderlich O, Williams R. Anti-Inflammatory and Skin-Hydrating Properties of a Dietary Supplement and Topical Formulations Containing Oligomeric Proanthocyanidins. *Skin pharmacology and physiology* (2007) 20(1):43-49. doi: 10.1159/000096171.

47. Shoji T, Masumoto S, Moriichi N, Ohtake Y, Kanda T. Administration of Apple Polyphenol Supplements for Skin Conditions in Healthy Women: A Randomized, Double-Blind, Placebo-Controlled Clinical Trial. *Nutrients* (2020) 12(4). doi: 10.3390/nu12041071.

48. Tsuchiya T, Fukui Y, Izumi R, Numano K, Zeida M. Effects of Oligomeric Proanthocyanidins (Opcs) of Red Wine to Improve Skin Whitening and Moisturizing in Healthy Women - a Placebo-Controlled Randomized Double-Blind Parallel Group Comparative Study. *European review for medical and pharmacological sciences* (2020) 24(3):1571-1584. doi: 10.26355/eurrev_202002_20215.

49. Heinrich U, Neukam K, Tronnier H, Sies H, Stahl W. Long-Term Ingestion of High Flavanol Cocoa Provides Photoprotection against Uv-Induced Erythema and Improves Skin Condition in Women. *The Journal of nutrition* (2006) 136(6):1565-1569. doi: 10.1093/jn/136.6.1565.

50. Mogollon JA, Boivin C, Lemieux S, Blanchet C, Claveau J, Dodin S. Chocolate Flavanols and Skin Photoprotection: A Parallel, Double-Blind, Randomized Clinical Trial. *Nutr J* (2014) 13:66. doi: 10.1186/1475-2891-13-66.

51. Fukagawa S, Haramizu S, Sasaoka S, Yasuda Y, Tsujimura H, Murase T. Coffee Polyphenols Extracted from Green Coffee Beans Improve Skin Properties and Microcirculatory Function. *Bioscience, biotechnology, and biochemistry* (2017) 81(9):1814-1822. doi: 10.1080/09168451.2017.1345614.

52. Tseng YP, Liu C, Chan LP, Liang CH. Coffee Pulp Supplement Affects Antioxidant Status and Favors Anti-Aging of Skin in Healthy Subjects. *J Cosmet Dermatol* (2021). doi: 10.1111/jocd.14341.

53. Heinrich U, Moore CE, De Spirt S, Tronnier H, Stahl W. Green Tea Polyphenols Provide Photoprotection, Increase Microcirculation, and Modulate Skin Properties of Women. *The Journal of nutrition* (2011) 141(6):1202-1208. doi: 10.3945/jn.110.136465.

54. Ito N, Seki S, Ueda F. The Protective Role of Astaxanthin for Uv-Induced Skin Deterioration in Healthy People-a Randomized, Double-Blind, Placebo-Controlled Trial. *Nutrients* (2018) 10(7). doi: 10.3390/nu10070817.

55. Phetcharat L, Wongsuphasawat K, Winther K. The Effectiveness of a Standardized Rose Hip Powder, Containing Seeds and Shells of Rosa Canina, on Cell Longevity, Skin Wrinkles, Moisture, and Elasticity. *Clinical interventions in aging* (2015) 10:1849-1856. doi: 10.2147/cia.S90092.

56. Yamashita E. The Effects of a Dietary Supplement Containing Astaxanthin on Skin Condition. *Food Style* (2006) 9(9).

57. Yamashita E. Cosmetic Benefit of Dietary Supplements Including Astaxanthin and Tocotrienol on Human Skin. *FOOD Style* (2002) 21(6).

58. Yoon HS, Cho HH, Cho S, Lee SR, Shin MH, Chung JH. Supplementating with Dietary Astaxanthin Combined with Collagen Hydrolysate Improves Facial Elasticity and Decreases Matrix Metalloproteinase-1 and -12 Expression: A Comparative Study with Placebo. *Journal of medicinal food* (2014) 17(7):810-816. doi: 10.1089/jmf.2013.3060.

59. Palombo P, Fabrizi G, Ruocco V, Ruocco E, Fluhr J, Roberts R, et al. Beneficial Long-Term Effects of Combined Oral/Topical Antioxidant Treatment with the Carotenoids Lutein and Zeaxanthin on Human Skin: A Double-Blind, Placebo-Controlled Study. *Skin pharmacology and physiology* (2007) 20(4):199-210. doi: 10.1159/000101807.

60. Kaminaka C, Yamamoto Y, Sakata M, Hamamoto C, Misawa E, Nabeshima K, et al. Effects of Low-Dose Aloe Sterol Supplementation on Skin Moisture, Collagen Score and Objective or Subjective Symptoms: 12-Week, Double-Blind, Randomized Controlled Trial. *The Journal of dermatology* (2020) 47(9):998-1006. doi: 10.1111/1346-8138.15428.

61. Tanaka M, Misawa E, Yamauchi K, Abe F, Ishizaki C. Effects of Plant Sterols Derived from Aloe Vera Gel on Human Dermal Fibroblasts in Vitro and on Skin Condition in Japanese Women. *Clinical, cosmetic and investigational dermatology* (2015) 8:95-104. doi: 10.2147/ccid.S75441.

62. Tanaka M, Yamamoto Y, Misawa E, Nabeshima K, Saito M, Yamauchi K, et al. Effects of Aloe Sterol Supplementation on Skin Elasticity, Hydration, and Collagen Score: A 12-Week Double-Blind, Randomized, Controlled Trial. *Skin pharmacology and physiology* (2016) 29(6):309-317. doi: 10.1159/000454718.

63. Vaughn AR, Clark AK, Notay M, Sivamani RK. Randomized Controlled Pilot Study of Dietary Supplementation with Turmeric or Herbal Combination Tablets on Skin Barrier Function in Healthy Subjects. *Journal of medicinal food* (2018) 21(12):1260-5. doi: 10.1089/jmf.2018.0015.

64. Asada K, Ohara T, Muroyama K, Yamamoto Y, Murosaki S. Effects of Hot Water Extract of Curcuma Longa on Human Epidermal Keratinocytes in Vitro and Skin Conditions in Healthy Participants: A Randomized, Double-Blind, Placebo-Controlled Trial. *J Cosmet Dermatol* (2019) 18(6):1866-1874. doi: 10.1111/jocd.12890.

65. Nagae M, Nagata M, Teramoto M, Yamakawa M, Matsuki T, Ohnuki K, et al. Effect of Porcine Placenta Extract Supplement on Skin Condition in Healthy Adult Women: A Randomized, Double-Blind Placebo-Controlled Study. *Nutrients* (2020) 12(6). doi: 10.3390/nu12061671.

66. Kim K, Sung J, Lee H, Ono T, Yonei Y. Effect of a Dietary Supplement Containing Porcine Placenta Extract on Skin Hydration: A Placebo-Controlled, Randomized, Double-Blind, Clinical Study. *Japanese Pharmacology and Therapeutics* (2018) 46:1023-1034.
